# Perioperative statin therapy in cardiac surgery: a meta-analysis of randomized controlled trials

**DOI:** 10.1186/s13054-016-1560-6

**Published:** 2016-12-05

**Authors:** Alessandro Putzu, Bruno Capelli, Alessandro Belletti, Tiziano Cassina, Enrico Ferrari, Michele Gallo, Gabriele Casso, Giovanni Landoni

**Affiliations:** 1 Department of Cardiovascular Anesthesia and Intensive Care, Cardiocentro Ticino, Via Tesserete 48, Lugano, Switzerland; 2 Department of Anesthesia and Intensive Care, IRCCS San Raffaele Scientific Institute, Via Olgettina 60, Milano, Italy; 3 Department of Cardiac Surgery, Cardiocentro Ticino, Via Tesserete 48, Lugano, Switzerland; 4 Vita-Salute San Raffaele University, Via Olgettina 58, Milano, Italy

**Keywords:** Cardiac surgery, Statins, Acute kidney injury, Atrial fibrillation, Myocardial infarction, Stroke, Mortality, Cardiac anesthesia, Intensive care

## Abstract

**Background:**

Several studies suggest beneficial effects of perioperative statin therapy on postoperative outcome after cardiac surgery. However, recent randomized controlled trials (RCTs) show potential detrimental effects. The objective of this systematic review is to examine the association between perioperative statin therapy and clinical outcomes in cardiac surgery patients.

**Methods:**

Electronic databases were searched up to 1 November 2016 for RCTs of preoperative statin therapy versus placebo or no treatment in adult cardiac surgery. Postoperative outcomes were acute kidney injury, atrial fibrillation, myocardial infarction, stroke, infections, and mortality. We calculated odds ratios (ORs) and 95% confidence intervals (CIs) using fixed-effects meta-analyses. Primary analysis was restricted to trials with low risk of bias according to Cochrane methodology, and sensitivity analyses examined whether the risk of bias of included studies was associated with different results. We performed trial sequential analysis (TSA) to test the strength of the results.

**Results:**

We included data from 23 RCTs involving 5102 patients. Meta-analysis of trials with low risk of bias showed that statin therapy was associated with an increase in acute kidney injury (314 of 1318 (23.82%) with statins versus 262 of 1319 (19.86%) with placebo; OR 1.26 (95%CI 1.05 to 1.52); *p* = 0.01); these results were supported by TSA. No difference in postoperative atrial fibrillation, myocardial infarction, stroke, infections, or mortality was present. On sensitivity analysis, statin therapy was associated with a slight increase in hospital mortality. Meta-analysis including also trials with high or unclear risk of bias showed no beneficial effects of statin therapy on any postoperative outcomes.

**Conclusions:**

There is no evidence that statin therapy in the days prior to cardiac surgery is beneficial for patients’ outcomes. Particularly, statins are not protective against postoperative atrial fibrillation, myocardial infarction, stroke, or infections. Statins are associated with a possible increased risk of acute kidney injury and a detrimental effect on hospital survival could not be excluded. Future RCTs should further evaluate the safety profile of this therapy in relation to patients’ outcomes and assess the more appropriate time point for discontinuation of statins before cardiac surgery.

**Electronic supplementary material:**

The online version of this article (doi:10.1186/s13054-016-1560-6) contains supplementary material, which is available to authorized users.

## Background

Postoperative complications after cardiac surgery are associated with higher morbidity and mortality, and increased costs [1]. Non-fatal complications are relatively common [2, 3], with the most important ones being acute kidney injury (AKI) [4, 5], atrial fibrillation (AF) [6, 7], myocardial infarction (MI), and stroke [8], while the overall 30-day mortality is approximately 3%.

Current American guidelines [9] highly recommend preoperative treatment with statins in all patients undergoing coronary artery bypass grafting (CABG), irrespective of their preoperative lipid profile, with rapid restoration of statin therapy after surgery. Discontinuation of statin treatment is not recommended before or after CABG in patients without side effects to therapy [9]. Therefore, nowadays more than half of patients scheduled for CABG receive perioperative statins [10] in compliance with present guidelines.

Moreover, knowledge of pleiotropic anti-inflammatory effects of statins [11, 12] has led to consider statins a potential therapy able to modulate the inflammatory response to cardiac surgery. In support of this assumption, several randomized controlled trials (RCTs), reporting on inflammatory markers and statin use in perioperative cardiac surgery, have demonstrated reduction in inflammatory cytokines [12]. In several retrospective non-randomized studies, preoperative statins have been associated with lower postoperative MI, mortality [13–17], AF [17, 18], neurological dysfunction [16], renal dysfunction [19], and infection [20]. However, the largest recently published RCTs show that perioperative statins do not prevent postoperative AF or myocardial damage and could be even associated with higher postoperative AKI [21, 22].

Due to the contrasting results and equivocal quality of evidence in the current literature, we performed a systematic review and meta-analysis of RCTs to examine the effects of perioperative statin therapy on postoperative AKI, AF, MI, stroke, infections, and mortality in adult cardiac surgical patients.

## Methods

We conducted a systematic review and meta-analysis of randomized trials, in compliance to the Cochrane methodology [23] and Preferred Reporting Items for Systematic Reviews and Meta-Analyses (PRISMA) guidelines [24], and according to a pre-published protocol on the PROSPERO database (CRD42016039509 [25]). A complete PRISMA 2009 checklist is provided in the supplementary material (Additional file 1). This study had no funding and authors did not have any conflicts of interest.

### Search strategy

Two trained investigators (AP, AB) independently searched PubMed, the Cochrane Central Register of clinical trials, and EMBASE (last updated on 1 November 2016) for appropriate articles. The full PubMed search strategy is presented in the supplementary material (Additional file 1). The search strategy was designed to include any RCT ever performed with perioperative statin therapy compared to control in adult humans in a cardiac surgery setting. No language restriction was enforced. References for eligible studies and identified reviews were searched by hand.

### Study selection

Records obtained from searches were first independently examined at an abstract level by two trained investigators (AP and AB). Following the initial abstract assessment, all identified studies were acquired as full-text. Eligible studies met the following criteria defined as patient, population or problem, intervention, comparison, outcomes and study design (PICOS): (1) population: adult cardiac surgery patients; (2) intervention: administration of perioperative statin therapy; (3) comparison intervention: placebo or no active intervention as control; (4) outcome: any primary or secondary outcome of the present systematic review (see subsequent text); and (5) study design: randomized controlled trial. The exclusion criteria were pediatric studies and overlapping populations. Two investigators (AP and AB) independently assessed selected studies for the final analysis, with eventual divergences finally resolved by consensus with a third author (GL).

### Data abstraction and study characteristics

Two authors (AP and AB) independently extracted data from studies and entered them into a predefined database. Discrepancies were identified and resolved through discussion with a third author (GL) if necessary. We collected potential sources of significant clinical heterogeneity such as study design, clinical setting, details of the case and control interventions, data on the predefined outcomes, and information necessary to assess risk of bias.

The primary outcomes were postoperative AKI, postoperative AF, postoperative MI, postoperative stroke, and postoperative infection. The secondary outcome was mortality at the longest available follow up. The outcomes were reported in the present review as per-author definition. If the data on the postoperative outcomes were absent or incomplete, missing data were requested from the corresponding authors of the study. The data extraction followed the intention-to-treat basis whenever possible.

### Assessment of quality of the included studies

We used the Cochrane approach [23, 26] to evaluate the methodological quality of each included trial (Additional file 1). Each trial was finally judged to be of low, unclear, or high risk of bias. The quality of the evidence for each outcome was summarized with the grading of recommendations assessment, development, and evaluation (GRADE) method [23, 26, 27].

### Statistical analysis

For each outcome, we calculated the odds ratio (OR) with 95% confidence intervals (CI). We reported the proportion of patients with the outcome in each group and the *p* value for the comparison between the groups. A *p* value <0.05 was considered significant. In the case of statistically significant ORs, we calculated the number needed to treat (NNT) or number needed to harm (NNH). The primary analysis of the present review was restricted to studies with low risk of bias, as suggested by the Cochrane Collaboration tool for assessing risk of bias [26].

Heterogeneity was explored by the Cochran *Q* statistic and characterized with *I* ^2^. We used a fixed-effect model for meta-analysis in the absence of significant heterogeneity, defined as a *p* value >0.10 and *I* ^2^ < 50%. In case of significant heterogeneity, we employed the random-effects model except if few trials dominated the available evidence or if significant publication bias was present, as random-effects meta-analysis in these contexts can give inappropriately high weight to smaller studies [23]. Two investigators (AP and AB) independently evaluated publication bias and small trials bias, analyzing a funnel plot and assessing the asymmetry in the funnel plot of trial size against treatment effect.

We performed sensitivity analyses for each outcome in order to assess the influence of risk of bias in the trials, including all eligible trials despite their risk of bias and including only trials with unclear or high risk of bias. In accordance with the Cochrane methodology [23], we performed sensitivity analysis for each outcome to investigate whether choice of summary statistic (OR, risk ratio (RR), risk difference (RD)) is critical to the results of the meta-analysis. We performed further sensitivity analysis for each outcome including only trials enrolling more than 200 patients or including only placebo-controlled studies.

Two authors independently evaluated the possibility of significant conflicts of interest within each study. They evaluated the funding of the study, the potential for authors’ conflicts of interests, the methodological quality of the study, and the positive/negative/indifferent findings of the study over statin. In case of possible or unclear industrial conflicts of interest among studies included in the analysis, we performed sensitivity analysis excluding them. The results of sensitivity analyses are reported only if significantly different from the primary analysis.

Post-hoc meta-regression was employed to examine the possible influence of length of preoperative therapy, proportion of CABG patients, trial size, and publication year on clinical outcomes in all eligible trials. Post-hoc subgroup analyses were performed on trials that included only statin-naïve patients, on trials enrolling mixed populations (statin-naïve and chronic statin therapy), and on trials that randomized patients on a postoperative statin regimen or not. Subgroup differences were tested using chi-square statistics [23]. The meta-analysis was performed using Review Manager (RevMan (Computer program), Version 5.3. Copenhagen: The Nordic Cochrane Centre, The Cochrane Collaboration, 2014).

Finally, to confirm the validity of our findings, we performed post-hoc trial sequential analysis (TSA) [28–30], with the intent of maintaining an overall 5% risk of type I error and a 20% risk of type II error, at a power of 80%. Relative risk reduction (RRR) or relative risk increase (RRI) for each outcome was derived from the literature in order to evidence a clinically meaningful difference (Additional file 1). We used the Copenhagen Trial Unit TSA software (version 1.0, http://www.ctu.dk/tsa).

## Results

### Study characteristics

**Fig. 1:**
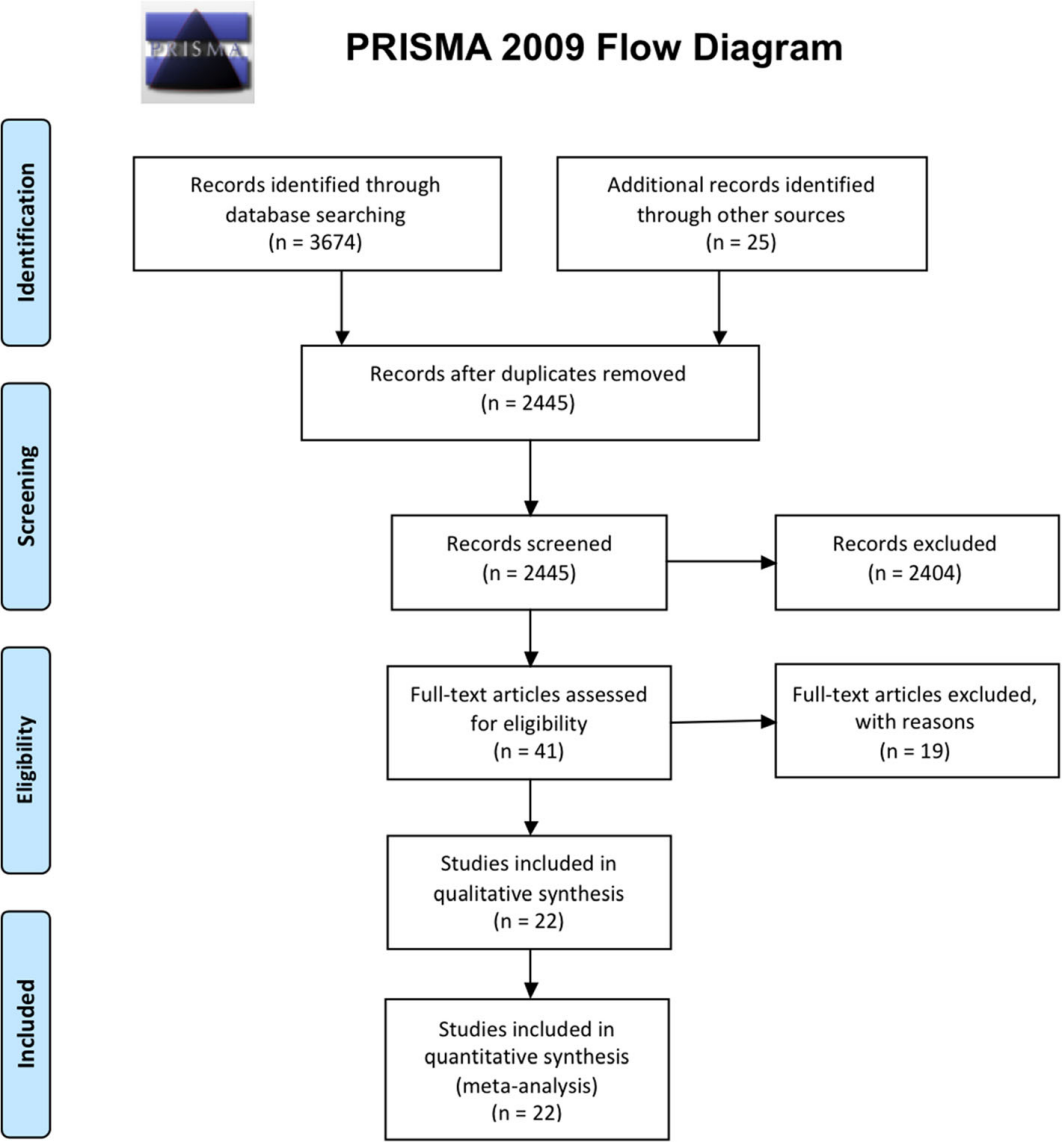
Study flow diagram. *PRISMA* Preferred Reporting Items for Systematic Reviews and Meta-Analyses

In total, 3699 references were examined. Major exclusions are presented in the supplementary data together with the reasons for exclusion (Additional file 1). Finally, 23 articles (5102 randomized patients) [21, 22, 31–51] were included in the analysis (Fig. 1).

Characteristics of the 23 trials are listed in Table 1 and Additional file 1. Eligible trials included from 30 to 1922 patients and were all single-center studies. In six cases we received data from the authors on further outcomes [21, 31, 38, 40, 42, 51].

**Table 1:** Characteristics of the trials included in the analysis

Trial	Journal	Cardiac surgery procedure	Number of patients	Statin	Statin regimen	Duration of preoperative therapy	Control	Patients naïve to statin therapy?	Outcomes for meta-analysis	Risk of bias
Almansob 2012 [31]	Arterioscler Thromb Vasc Biol	Non coronary cardiac surgery	132	Simvastatin 20 mg	5–7 days preop and from day 2 postop (no day 0)	5–7 days	No treatment	NR	AF, MI, S, M	High
Baran 2012 [32]	Stem Vell Rev and Rep	CABG	60	Atorvastatin 40 mg	2 weeks preop and postop (no day 0)	2 weeks	Placebo	Yes (>3 months)	AKI, AF, MI, S, M	High
Berkan 2009 [33]	Thorac Cardiov Surg	CABG	46	Fluvastatin 80 mg	3 weeks preop	3 weeks	Placebo	Yes (>1 year)	MI	High
Billings 2016 [21]	JAMA	CABG (49%), valve, or ascending aortic surgery	615	Atorvastatin 40 mg	Bid day 1^b^, qd day 0, and qd postop	2 days	Placebo	No (Naïve 32%)	AKI, AF, MI, S, M, I	Low
Caorsi 2008 [34]	Eur Cytokine Netw	On-pump CABG	43	Pravastatin 40 mg	2 days preop, 1 hour after CPB, and 7 days postop	2 days	No treatment	NR	AF	High
Carrascal 2016 [51]	J Arrhythm	Valve surgery	90	Atorvastatin 40 mg	7 days preop and 7 days postop	7 days	No treatment	Yes	AKI, AF, MI, S, M	High
Castaño 2015 [35]	J Cardiovasc Surg	On-pump CABG	30	Pravastatin 80 or 40 mg	2 hours before surgery	1 days	Placebo	No (Naïve 0%)	AF, MI, M	Unclear
Chello 2006 [36]	Crit Care Med	On-pump CABG	40	Atorvastatin 20 mg	3 weeks preop	3 weeks	Placebo	Yes (>1 year)	AKI, AF, MI, S, M, I	Unclear
Christenson 1999 [37]	Eur J Cardiothorac Surg	On-pump CABG	77	Simvastatin 20 mg	4 weeks preop	4 weeks	No treatment	NR	AKI, MI, M, I	High
Dehghani 2015 [38]	J Cardiovasc Pharmacol Ther	Valve surgery	58	Atorvastatin 40 mg	3 days preop and 5 days postop	3 days	Placebo	Yes	AF, MI, S, M	Unclear
Ji 2009 [39]	Circ J	Off-pump CABG	140	Atorvastatin 20 mg	7 days preop	7 days	Placebo	Yes	AF, MI, S, M	High
Mannacio 2008 [40]	J Thorac Cardiovasc Surg	On-pump CABG	200	Rosuvastatin 20 mg	7 days preop	7 days	Placebo	Yes (>1 month)	AKI, AF, MI, S, M, I	High
Melina 2009 [41]^a^	Eur Heart J	On- off-pump CABG	632	Atorvastatin 40 mg	NR	NR	Placebo	NR	AF	High
Park 2016 [42]	Intensive Care Med	Valve surgery	200	Atorvastatin 40 mg	Bid day 1, qd day 0, and qd 3 days postop^b^	2 days	Placebo	Yes	AKI, AF, MI, S, M	Unclear
Patti 2006 [43]	Circulation	On-pump cardiac surgery (CABG 79%)	200	Atorvastatin 40 mg	7 days preop and postop until discharge (no day 0)	7 days	Placebo	Yes (>1 year)	AF, MI, M	High
Prowle 2012 [44]	Nephrology	On-pump cardiac surgery (CABG 57%)	100	Atorvastatin 40 mg	Days 0 and 3 days postop	1 days	Placebo	No (Naïve 30%)	AKI, M	Low
Song 2008 [45]	Am Heart J	Off-pump CABG	124	Atorvastatin 20 mg	3 days preop, after surgery, and 30 days postop	3 days	No treatment	Yes	AF, MI, S	High
Spadaccio 2010 [46]	J Cardiovasc Pharmacol	On-pump CABG	50	Atorvastatin 20 mg	3 weeks preop	3 weeks	Placebo	Yes (>1 year)	AKI, AF, MI, S, M, I	Unclear
Sun 2011 [47]	Int Heart J	On-pump CABG	100	Atorvastatin 20 mg	7 days preop	7 days	Placebo	Yes (>14 days)	AF, MI	High
Tamayo 2009 [50]	J Thorac Cardiovasc Surg	On-pump CABG	44	Simvastatin 20 mg	3 weeks preop	3 weeks	No treatment	Yes (>3 weeks)	AF, M	High
Vukovic 2011 [48]	Perfusion	On-pump CABG	57	Atorvastatin 20 mg	3 weeks preop	3 weeks	Placebo	Yes (>1 year)	AF, MI, M, I	High
Youn 2011 [49]	Korean J Thorac Cardiovasc Surg	Off-pump CABG	142	Rosuvastatin 20 mg	Bid day 1, qd day 0	2 days	No treatment	No (Naïve 45%)	MI, M	High
Zheng 2016 [22]	N Engl J Med	On-pump CABG (42%), off-pump CABG (43%), AVR, AVR + CABG	1922	Rosuvastatin 20 mg	1–8 days preop and 5 days postop	8 days	Placebo	No (Naïve 66%)	AKI, AF, MI, S, M, I	Low

^a^Abstract-only publication. ^b^Patients on chronic statin therapy received study drug only on day 0 and day 1, resuming chronic statin therapy on postoperative day 2. *CABG* coronary artery bypass grafting, *AVR* aortic valve replacement, *CPB* cardiopulmonary bypass, *preop* preoperative regimen, *postop* postoperative regimen, *day 0* the morning of the day of surgery, *qd* once a day, *bid* twice a day, *AF* atrial fibrillation, *AKI* acute kidney injury, *MI* myocardial infarction, *I* infection, *M* mortality, *NR* not reported

All trials included elective cardiac surgery patients and the most represented procedure was CABG, performed in 78.01% of the patients.

Statins were administered preoperatively in all trials, with the length of treatment ranging from 1 to 28 days (median 7 days). The total duration of statin treatment varied from 2 to 33 days; the variety of statin doses and regimens are shown in Table 1. The randomized treatment was also administered postoperatively in 8 trials [22, 34, 35, 38, 42, 44, 45, 51]. Atorvastatin (20 to 80 mg) was administered in 14 trials, Simvastatin 20 mg in 3 trials, Rosuvastatin 20 mg in 2 trials, Fluvastatin 80 mg in 1 trial, and Pravastatin 40 mg in 1 trial. In one case the statin regimen was not specified [41]. Placebo was administered as control in 16 trials and in 7 trials there was no intervention administered as control [31, 34, 37, 45, 49–51].

Ten trials reported postoperative data on AKI (3354 patients), 19 trials on AF (4737 patients), 19 trials on MI (4283 patients), 12 trials on stroke (3631 patients), 7 trials on infections (2961 patients), and 18 trials on mortality (4157 patients).

**Fig. 2:**
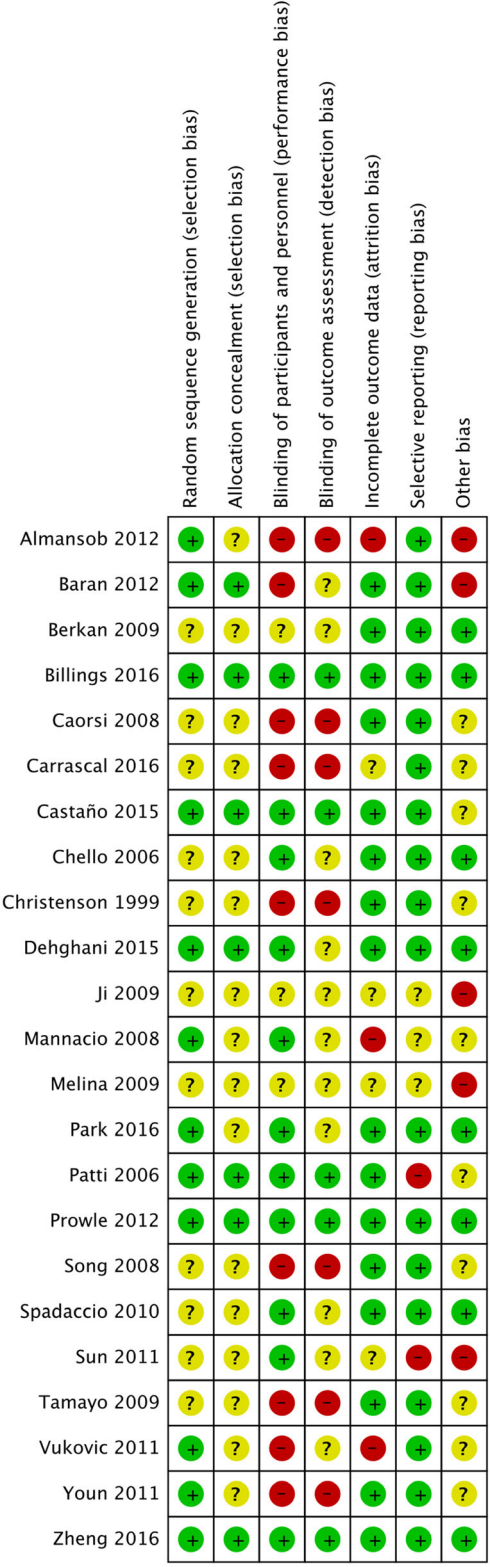
Risk of bias summary: review authors' judgments about each risk of bias item for each included study. *Green* circles indicate low risk of bias, *yellow* circles indicate unclear risk of bias, and *red* circles indicate high risk of bias

Three trials were judged at low risk of bias in all bias domains [21, 22, 44]. Five trials were scored as having unclear risk of bias [35, 36, 38, 42, 46] and 15 trials were at high risk of bias (Fig. 2 and Additional file 1). The overall quality of evidence according to GRADE was reported for each outcome (Table 2 and Additional file 1).

**Table 2:** Postoperative outcomes: effects of perioperative statin therapy versus control

Postoperative outcome	Number of trials	Number of patients	OR (95% CI)	*P* value	Trial sequential analysis	Level of evidence
Acute kidney injury						
Trials with low risk of bias	3	2637	1.26 (1.05, 1.52)	*0.01*	*Conclusive: harmful of statins*	High
All trials	10	3354	1.18 (0.99, 1.41)^a^	0.06	Inconclusive	Low
Atrial fibrillation						
Trials with low risk of bias	2	2537	1.08 (0.90, 1.30)	0.40	*Conclusive: futility of statins*	Moderate
All trials	19	4737	0.80 (0.70, 0.91)^a^	*0.001*	Inconclusive	Very low
Myocardial infarction						
Trials with low risk of bias	2	2537	0.97 (0.71, 1.33)	0.87	*Conclusive: futility of statins*	Moderate
All trials	18	4151	0.92 (0.69, 1.23)^a^	0.56	*Conclusive: futility of statins*	Very low
Stroke						
Trials with low risk of bias	2	2537	1.25 (0.58, 2.70)	0.56	*Conclusive: futility of statins*	Low
All trials	11	3499	1.09 (0.60, 2.00)	0.77	*Conclusive: futility of statins*	Very low
Infections						
Trials with low risk of bias	2	2537	0.80 (0.60, 1.07)	0.14	Inconclusive	Low
All trials	7	2724	0.78 (0.59, 1.04)	0.09	*Conclusive: futility of statins*	Very low
Mortality						
Trials with low risk of bias	3	2637	3.84 (0.95, 15.55)	0.06	Inconclusive	Low
All trials	18	4157	1.92 (0.79, 4.66)	0.15	Inconclusive	Very low

^a^Evidence of publication bias in favor of statins. Italics indicate statistical significance. *OR* odds ratio, *CI* confidence interval

### Acute kidney injury

**Fig. 3:**
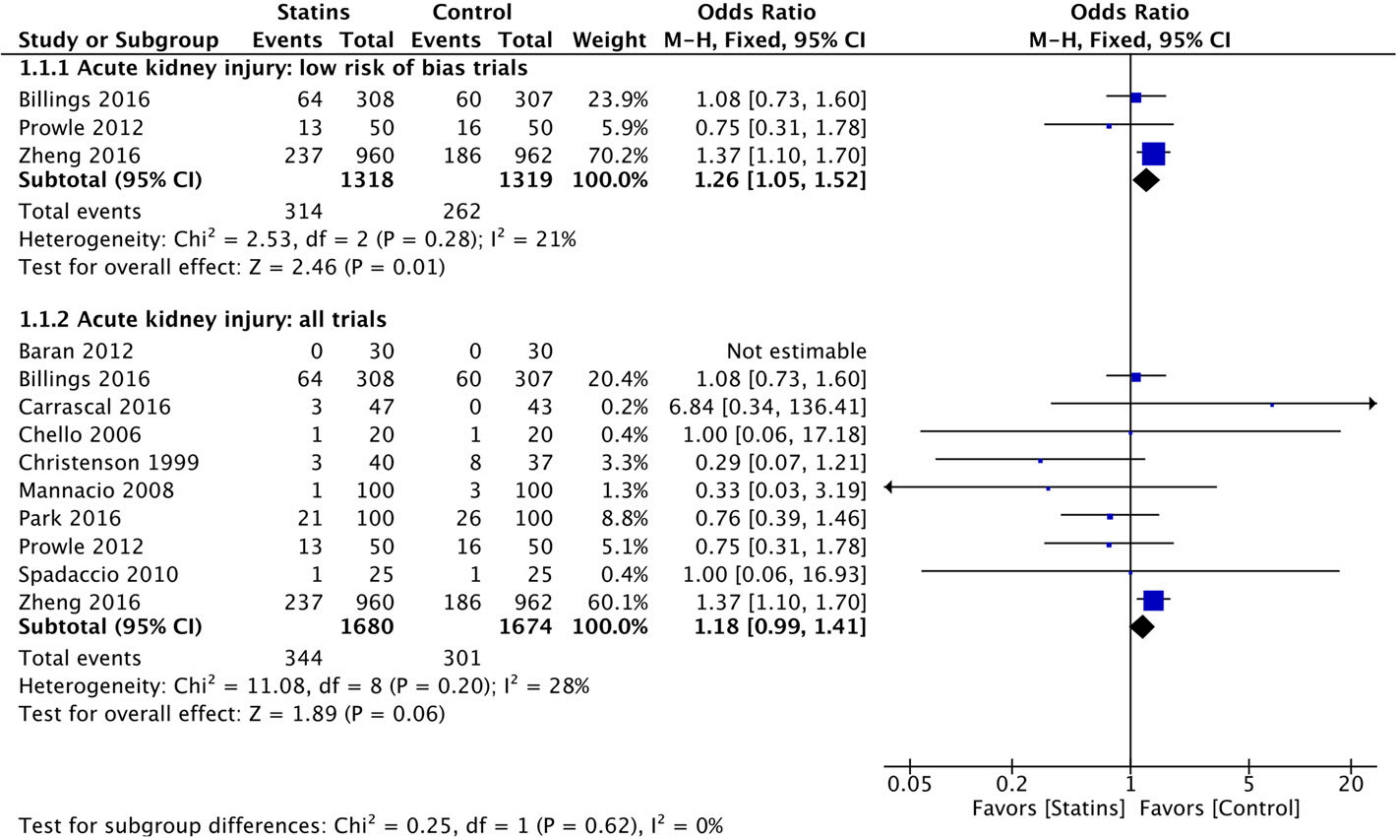
Postoperative acute kidney injury (AKI). Forest plot shows postoperative AKI in patients with perioperative statin therapy vs. control

When including trials with low risk of bias, the administration of perioperative statins was associated with increased incidence of postoperative AKI as compared with placebo (314 of 1318 patients (23.82%) in the statin group versus 262 of 1319 patients (19.86%) in the placebo group; OR 1.26 (95% CI 1.05–1.52); *p* = 0.01; NNH 25) (Fig. 3). The results are supported by the TSA, which showed firm evidence for a 25% RRI (Additional file 1). The overall quality of evidence was high according to GRADE.

The quantitative results of the systematic review are displayed below and in Table 2 and supplementary material (Additional file 1).

When including all the eligible trials despite the risk of bias, statin therapy was associated with a non-significant difference in AKI versus control (OR 1.18 (95% CI 0.99–1.41]; *p* = 0.06) (Fig. 3). Nonetheless, the latter analysis was characterized by possible small-study publication bias and on sensitivity analyses the incidence of AKI was higher with statin therapy (Additional file 1).

### Atrial fibrillation

**Fig. 4:**
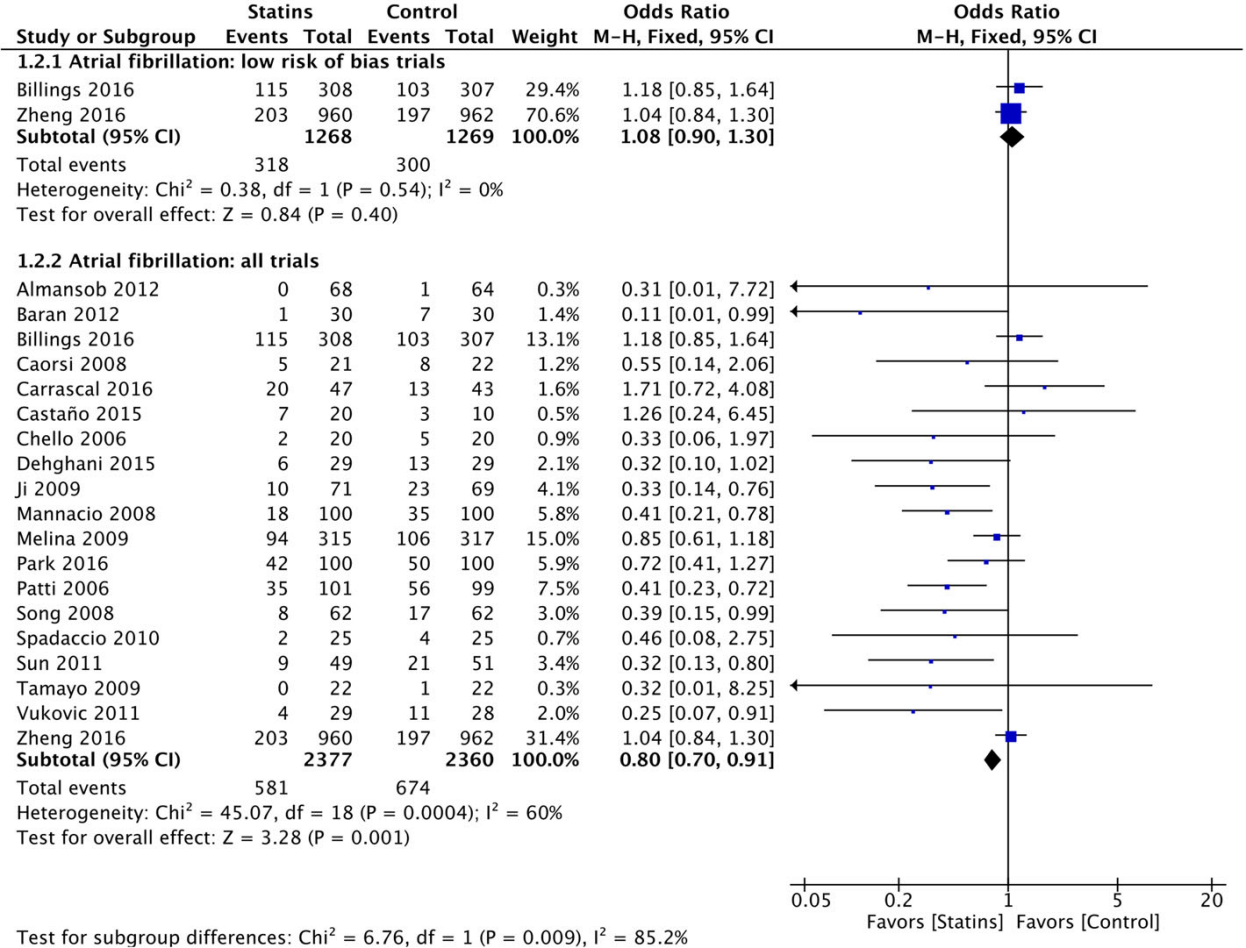
Postoperative atrial fibrillation (AF). Forest plot shows postoperative AF in patients with perioperative statin therapy vs. control

There was no difference in the rate of postoperative AF in trials with low risk of bias (318 of 1268 (25.07%) in the statin group and 300 of 1269 (23.64%) in the placebo group, OR 1.08 (95% CI 0.90–1.30); *p =* 0.40) (Fig. 4) and the TSA showed futility of the statin treatment when assuming an RRR of 20% (Additional file 1). The overall quality of evidence was moderate.

On the contrary, the analysis that also included trials with high and unclear risk of bias showed a lower incidence of AF among patients allocated to statins (Fig. 4), but TSA did not confirm the findings. There was significantly high heterogeneity (*p* for heterogeneity = 0.0004, *I* ^2^ 60%) and important small-study publication bias; on sensitivity analyses there was no difference in AF between statins and control when including all eligible trials despite their risk of bias (Additional file 1).

### Myocardial infarction

**Fig. 5:**
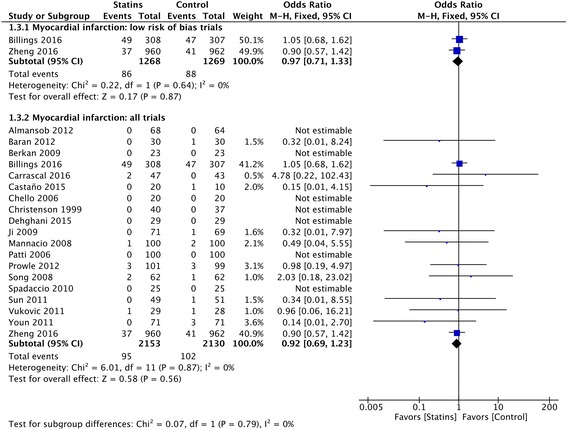
Postoperative myocardial infarction (MI). Forest plot shows postoperative MI in patients with perioperative statin therapy vs. control

The rate of postoperative MI did not change significantly between groups among trials with low risk of bias (86 of 1268 (6.78%) in the statin group versus 88 of 1269 (6.93%) in the placebo group; OR 0.90 (95% CI 0.57–1.42)) (Fig. 5) and TSA showed futility of the statin treatment when assuming an RRR of 30% (Additional file 1). The overall quality of evidence was moderate.

When including all trials regardless of the risk of bias, statin therapy was not associated with a difference in postoperative MI versus control (Fig. 5); publication bias was present (Additional file 1).

### Stroke

There was no difference in the rate of postoperative stroke in trials with low risk of bias (15 of 1268 (1.18%) in the statin group versus 12 of 1269 (0.95%) in the placebo group; OR 1.25 (95% CI, 0.58–2.70)) and TSA showed futility of the statin treatment when assuming an RRR of 80%. The overall quality of evidence was moderate. Including all trials, statin therapy was not associated with a difference in the rate of postoperative stroke (Additional file 1).

### Infections

There was no difference in the rate of postoperative infections between the statin and placebo groups in trials with low risk of bias (88 of 1268 (6.94%) in the statin group versus 108 of 1269 (8.51%) in the placebo group; OR 0.80 (95% CI 0.60, 1.07)); on meta-analysis including also trials with higher risk of bias there was no significant difference between groups (Additional file 1). TSA showed futility of the statin treatment only when including all the eligible trials despite their risk of bias (Additional file 1). The overall quality of evidence was low.

### Mortality

**Fig. 6:**
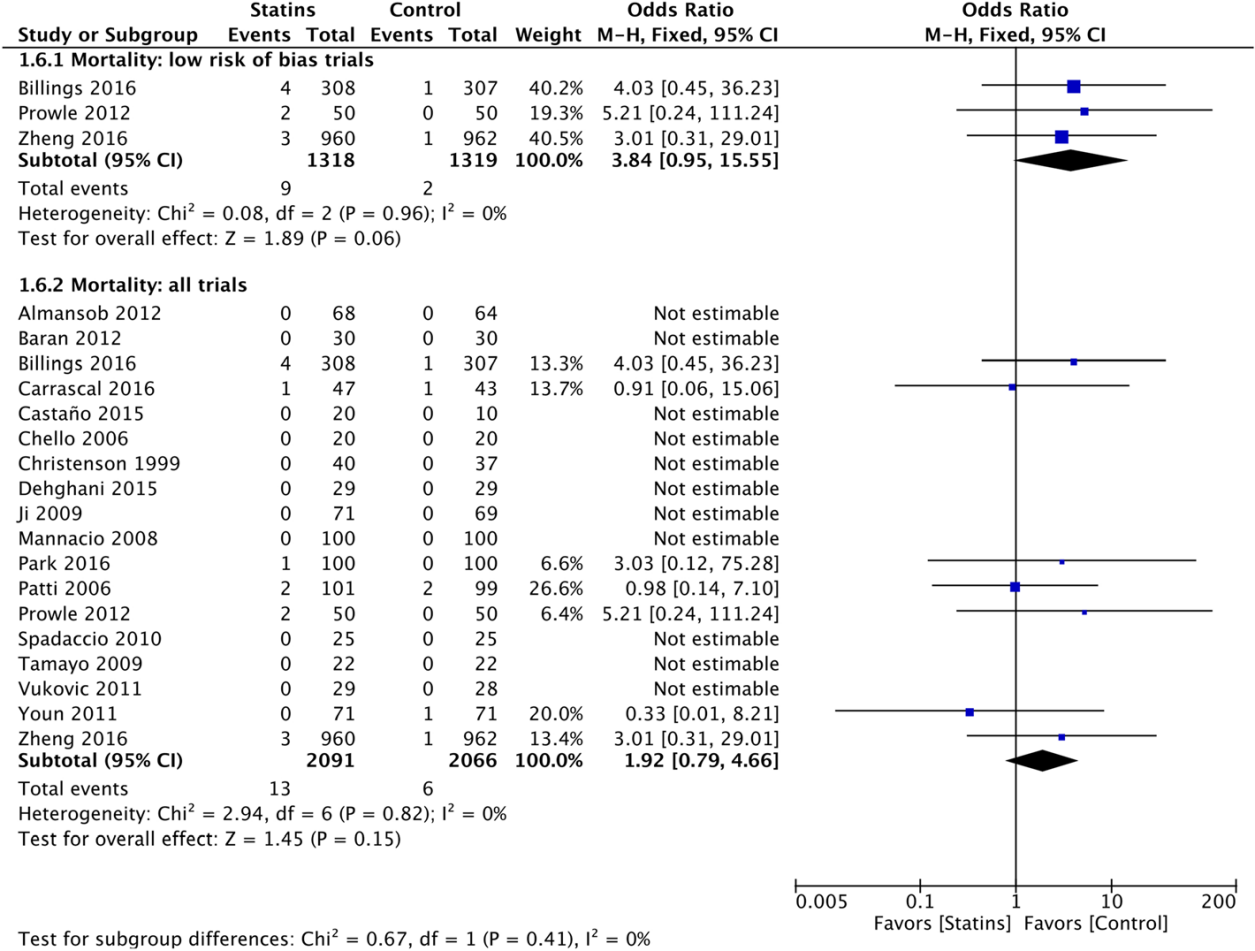
Postoperative mortality. Forest plot shows postoperative short-term mortality in patients with perioperative statin therapy vs. control (in-hospital or 30-day follow up)

The administration of perioperative statins was not associated with a significant difference in hospital mortality in trials with low risk of bias (9 of 1318 (0.68%) in the statin group versus 2 of 1319 (0.15%) in the placebo group; OR 1.26 [95% CI, 1.05-1.52]; *p* = 0.06) (Fig. 6); TSA did not support this as firm evidence. The overall quality of evidence was low. The sensitivity analysis showed a small increase in hospital mortality in the statin group when changing the summary statistic (RD 0.01 (95%CI 0.00–0.01); *p* 0.04) (Additional file 1). There was no difference in short-term mortality when including all eligible trials despite their risk of bias (Fig. 6).

### Clinical outcomes and perioperative statin regimen

Meta-regression analysis of trials with low risk of bias did not identify possible relationships between the length of the preoperative regimen and clinical outcomes. Meta-regression including all trials revealed a possible relationship between the length of the preoperative regimen and AF, with patients on longer preoperative regimens having a lower incidence of AF then patients on shorter regimens (*p* = 0.024). However, the latter analysis was driven mainly by the results of trials with unclear and high risk of bias (Additional file 1).

Subgroup analysis including all trials addressing the effect of the presence or lack of a postoperative regimen identified a significant subgroup difference for AKI (chi-squared 4.68, *p* = 0.03) and AF (chi-squared 19.21, *p* < 0.0001), suggesting a better outcome in patients not taking postoperative statins. However, the analysis has important limitations, as few trials did not administer postoperative therapy and all these trials had high or unclear risk of bias (Additional file 1).

### Clinical outcomes in patients on chronic statin therapy or statin-naïve patients

Among trials with low risk of bias, the only available outcome to estimate was AF (2 trials, 2537 patients) with no significant difference between statin-naïve patients or those on chronic therapy (chi-squared 0.05, *p* = 0.82). When including all the trials (19 trials, 4218 patients), there were no significant differences between trials including statin-naïve or mixed populations, except for AF (chi-squared 5.69, *p* = 0.02). However, this analysis should be interpreted with caution, because all the trials enrolling only statin-naïve patients had high or unclear risk of bias (Additional file 1).

### Clinical outcomes and CABG surgery

Meta-regression analysis did not reveal significant correlation between the proportion of patients undergoing CABG and the log-OR of any postoperative clinical outcomes analyzed, even when including trials with higher risk of bias (Additional file 1).

### Effect of higher risk of bias, publication year, trial size, and publication bias on clinical outcomes

Meta-regression including all trials demonstrated the influence of trial size (*p* = 0.02) and publication year (*p* < 0.01) on postoperative AF and AKI (Additional file 1). This underlines that: (1) AKI and AF rates changed over years, with results from older studies in favor of statins; and (2) AKI and AF rates changed in relation to trial sample size, with results from smaller trials in favor of statins.

The presence of possible publication bias was suggested when assessing the asymmetry of the funnel plot including all the eligible trials; in particular, publication bias was evident in AKI, AF, and MI (see previous), suggesting that small studies tended to report larger treatment effects in favor of statin therapy than larger studies did.

Meta-analysis including trials with high and unclear risk of bias (*n* = 20, 2465 patients) showed lower incidence of AF in the statin group and no significant difference in other postoperative outcomes (Additional file 1).

## Discussion

This study determined the effect of perioperative statin therapy on several postoperative outcomes in patients undergoing cardiac surgery. Our primary analysis including trials with low risk of bias showed that perioperative statin therapy was associated with a significantly higher incidence of AKI, whereas no other beneficial or detrimental effects on AF, MI, stroke, and infections were highlighted; a possible negative effect of statins on hospital mortality could not be ruled out. Moreover, our systematic review suggests that there is significant publication bias in favor of statin therapy when including all trials, as small studies and earlier studies, mostly with lower methodological quality and higher risk of bias, appear to have overestimated the beneficial effect of statins.

Statin administration is a cornerstone in lipid-lowering therapy and in prevention of cardiovascular problems [52], and after CABG surgery [53]. However, our systematic review highlights some important concerns involving the administration of this therapy in the days prior to cardiac surgery, in such patients undergoing major surgery at the risk of critical illness.

For many years, statin treatment was considered an attractive therapy for reducing AKI following cardiac surgery [54], an idea mainly based on retrospective data [16, 55–59], and according to this hypothesis, some large RCTs have been performed to test whether statins effectively decrease postoperative AKI [21, 42]. However, RCTs support the lack of a kidney-protective effect [21, 22, 42, 60, 61], as do the most recent systematic reviews [17, 60]. The largest RCT performed so far showed that rosuvastatin therapy resulted in a significantly higher rate of AKI and higher plasma creatinine levels compared to placebo at 48 hours after cardiac surgery [22]. Similarly, the second largest RCT published by Billings et al. [21], showed a non-significant trend in favor of placebo and a possible detrimental effect of statin therapy in the small subgroup of statin-naïve patients with chronic kidney disease. The authors suggested that the hypothetical association between preoperative use of statins and decreased postoperative AKI is inconsistent, suggesting that selection bias for statin use, variable effects of treatment, and disparate patient populations could have affected the results of prior retrospective trials attributing beneficial renal effects to statins.

There is still much to be learnt about the mechanisms of the possible negative effects of statin therapy on renal function, and a class effect in patients undergoing cardiac surgery cannot be ruled out. Mitochondrial dysfunction related to statins is a well-known pathological event that is frequently implicated in muscle adverse events; statins could promote oxidation and apoptosis, and unmask silent mitochondrial defects, leading to overall cellular energy imbalance [62]. It can be argued that mitochondrial dysfunction can be deleterious even in organs other than muscles, such as the kidneys, but no studies have explored this. Other potential mechanisms may include myoglobin nephropathy secondary to statin-induced rhabdomyolysis, possibly aggravated by a higher statin blood level due to drug interactions [62], and insulin resistance/aggravation of diabetes [62, 63].

According to high-quality evidence, there is no significant difference in the postoperative incidence of AF. On the other hand, we found conflicting results when including trials with higher risk of bias, but this analysis was characterized by significant small-study publication bias and significantly high heterogeneity. The results of larger RCTs [21, 22] conflict with those of several smaller RCTs [36, 39, 40, 43, 50, 64, 65], as smaller studies suggested that perioperative statin therapy, as compared with control, halved the incidence of postoperative AF. The assessment of postoperative AF could be biased due to several factors; for instance, continuous electrocardiogram monitoring during the study, definition of postoperative AF, and blinding of the personnel. In the largest RCT performed so far with postoperative AF as the primary endpoint, the incidence of postoperative AF did not differ significantly between patients receiving Rosuvastatin and those receiving placebo [22].

There was no significant difference in postoperative MI in association with perioperative statins. In the largest RCTs performed so far there was no difference in postoperative MI [21, 22] and myocardial injury, defined as difference in postoperative creatinine kinase-myocardial band (CK-MB) [21] and troponin I [22] release. In addition, we found no difference in postoperative stroke, another crucial cardiovascular complication associated to severe morbidity.

Statins have been thought to decrease postoperative infection [66], but our analysis ruled out a possible role in this field, as was also shown in other trials in a critical care setting, in which statin therapy had no effects on the progression of infection and mortality [67, 68].

According to randomized evidence, perioperative statins do not decrease short-term mortality, although an increase in hospital mortality among our population with low risk of bias could not be excluded. Future RCTs should explore this field, with particular attention to long-term mortality. Interestingly, the results of our systematic review are not in accordance with findings of previous observational studies [69] and meta-analyses including non-randomized studies and small randomized studies [16, 17], in which the authors describe a clear short-term mortality benefit mediated by perioperative statins compared to control; however, the retrospective design and the high risk of bias of the trials included make these results inconsistent.

With a total of 23 randomized trials and a cumulative patient cohort of 5102 patients, this was the largest meta-analysis of RCTs performed so far. The largest previous meta-analysis of RCTs included only 17 trials and 2138 patients and found no difference in any postoperative outcomes except for AF. However, the results of the aforementioned review were mainly driven by data with high risk of bias and did not include the two largest recently performed trials with low risk of bias trials [21, 22].

The included trials with low risk of bias were placebo-controlled, recruiting 2637 patients, 43% already on statin therapy, who underwent CABG surgery in about 78% of the cases. Current guidelines suggest that all patients undergoing CABG should receive or continue statin therapy, unless it is contraindicated, and statin discontinuation is not recommended before or after CABG because of possible harmful effects. In light of the results of the present systematic review and of the recently published high-quality trials [21, 22], the class and level of evidence of these recommendations should be revised, as current randomized evidence does not support the broad use of statin therapy in the perioperative period to improve patients’ outcomes. However, even if the majority of patients included in our analysis underwent CABG, we cannot rule out a possible class effect of statin therapy, although our meta-regression and the larger published trials [21, 22] did not suggest subpopulation effects.

In the authors’ opinion, this meta-analysis would support a neutral effect of perioperative statin therapy or perhaps weak evidence of a clinically significant detrimental effect on patients’ outcomes. The exact time-point for interruption of preoperative statin therapy in patients already taking a statin should be further evaluated. Billings and colleagues randomized patients on chronic statin therapy to intervention only on the day of the surgery and on the first postoperative day [21]. On the other hand, Zheng and colleagues interrupted statin therapy during the 8 days before surgery, with about two thirds of the patients having therapy interrupted during the 4 days before surgery [22]. However, the length of perioperative statin regimen varies among trials and no recommendations could be made.

### Strengths and limitations

One of the preferable meta-analytical strategies is to restrict the primary analysis to studies at low risk of bias [23, 26]. The choice should be based on the balance between the potential for bias and the loss of precision when studies at high and unclear risk of bias are excluded [26]. Among the randomized literature, we found significant small-study publication bias and serious differences in risk of bias within studies. We must recognize that publication bias is common in peer-reviewed journals and particularly in critical care medicine [70], because positive studies are easier and more attractive to publish than neutral or negative studies [71], and small trials are more likely to report larger beneficial effects than large trials, which could be partly explained by the lower methodological quality in smaller trials [70]. It is noteworthy that the largest trials with low risk of bias performed so far on the topic had neutral or negative results from analysis of the use of perioperative statins in cardiac surgery patients [21, 22]. To this end, we think that our per-protocol primary analysis, including only randomized placebo-controlled trials with low risk of bias, could have limited this problem. However, only three trials with low risk of bias with a total of 2637 patients have been published so far and included in our primary analysis.

There is some degree of variability in the nature of statin therapy, because statins may be administered using different strategies, some of which may be more effective than others; for example, there is non-randomized evidence to support an increased rate of AKI among patients taking high-potency statins, with the strongest rate in the first 4 months after initiation of treatment [72]. We did not perform subgroup analysis of different types of perioperative statin regimens, because almost all the trials administered different statin doses and formulations for different lengths of time, and the analysis would have been biased by results of trials with higher risk of bias. Finally, our mortality analysis included short-term mortality (in-hospital and 30-day mortality), addressing the fact that mortality should be assessed after longer follow up, in order to evidence the long-term effects of the interventions.

### Future directions

This systematic review synthesized evidence from RCTs and may help the execution of future clinical studies assessing the exact time point for interruption of preoperative statin therapy in patients already taking statins. Further RCTs should systematically evaluate the relationship between postoperative outcomes and variables related to the patient (e.g., chronic kidney disease, statin-naïvety), to the cardiac disease (e.g., coronary artery disease), to the surgical procedure (e.g., off-pump versus on-pump surgery), and to the specific statin regimen.

## Conclusions

Our results suggest that perioperative statin therapy is not protective against postoperative AF, MI, stroke, or infection. Instead, statins might be associated with higher postoperative AKI and a possible negative effect on short-term survival could not be excluded. Open questions on patient population, preexisting chronic disease, and length and dose of the treatment need to be clarified by further high-quality RCTs, assessing the more appropriate time point for discontinuation of statins before cardiac surgery.

## Additional files

Additional file 1: Supplementary material. (PDF 2.18 mb)

## Abbreviations

AF: atrial fibrillation
AKI: acute kidney injury
CABG: coronary artery bypass grafting
CI: confidence interval
MI: myocardial infarction
NNH: number needed to harm
NNT: number needed to treat
OR: odds ratio
PRISMA: Preferred Reporting Items for Systematic Reviews and Meta-Analyses
RCT: randomized controlled trial
RRI: relative risk increase
RRR: relative risk reduction
TSA: trials sequential analysis
