# Perioperative Statin Therapy and the Outcomes of Cardiac Surgery

**DOI:** 10.1186/s13054-016-1560-6

**Published:** 2016-12-05

**Authors:** Chunxia Shi, Nicole A. Weiss, Hong Liu

**Affiliations:** 1 Department of Anesthesiology, Chinese Academy of Medical Sciences Fuwai Hospital, Tsinghua University and Peking Union Medical College, National heart center, Beijing, China; 2 Department of Anesthesiology and Pain Medicine, University of California Davis Health, Sacramento, USA.

## Abstract

**Aim of review:**

Since the discovery in 1976, statins have been widely used in various patient populations, however, the decision on whether to initiate statins in the perioperative setting remains controversial. This article discusses perioperative statin therapy in patients undergoing cardiac surgery.

**Methods:**

In this review, we searched literature of the past 2 decades in databases of Embase, PubMed, the Cochrane Library and Science Citation Index, and focused on risks and benefits of perioperative statin therapy in patients undergoing cardiac surgery.

**Recent findings:**

While initial studies demonstrated their lipid-lowering effects, recent studies have focused on the pleiotropic effect; an effect which improves endothelial function, reduces vascular inflammation, stabilizes atherosclerotic plaques, inhibits oxidative stress and prevents vascular remodeling. These studies also brought to light the potential benefits of statins in the perioperative setting. In 2014, the American College of Cardiology (ACC) and American Heart Association (AHA) issued a class I recommendation to continue perioperative statins in those already receiving therapy. Recent studies have suggested that statins may also benefit high risk patients undergoing cardiac surgery.

**Conclusion:**

Most studies have shown that perioperative statin therapy can reduce incidence of major cardiovascular events, including myocardial infarction, atrial fibrillation, infection, stroke and death for patients undergoing cardiac surgery. (Funded by the University of California Davis Health Department of Anesthesiology and Pain Medicine, and the National Institutes of Health (NIH)).

As life expectancy increases, more high-risk patients with multiple comorbidities undergo surgical procedures. These patients will undoubtedly have increased risk of perioperative morbidity and mortality. With technological advances, the perioperative complications of cardiac surgery have been dramatically reduced; however, the morbidity and mortality remain high. The ASCERT trial found the mortality rate in elderly patients (>65 years) to be 8.1% at 1 year (1). Another study quoted the complication rate of cardiac surgery to be as high as 54% (2). These complications can often result in a reduced quality of life, a shorter lifespan and a significant increase in medical spending. Reducing perioperative complications for the patients undergoing cardiac surgery is pivotal to improving patient outcomes.

Discovered in 1976, statins are inhibitors of 3-hydroxy-3-methylglutaryl-coenzyme A (HMG CoA) reductase. They act to lower plasma levels of low-density lipid cholesterol (LDL-C) and have become a corner stone in the treatment of hyperlipidemia. While the lipid-lowering abilities of statins are well established, it is the pleiotropic effect of statins that has recently received attention. The pleiotropic effect has been shown to improve endothelial function, reduce vascular inflammation, stabilize atherosclerotic plaques, inhibit oxidative stress and prevent vascular remodeling.

The benefits of statin therapy in patients with cardiovascular disease have been well documented and it has been suggested that patients undergoing cardiac surgery may also benefit from the pleiotropic properties (3,4). Studies have demonstrated that perioperative statin therapy decreases postoperative complications, including atrial fibrillation (AF), myocardial infarction (MI), vascular thrombosis, acute kidney injury (AKI) and infection. It has also been shown to reduce patient mortality and shorten the length of hospital stay (LOS) in patients undergoing cardiac surgery (Table 1) (5-8). Despite these benefits, the initiation of statin therapy perioperatively remains controversial due to potential adverse effects. This review focuses on the risks and benefits of statin use in cardiovascular surgery.

## Pharmacology of Statins

### Lipid-Lowering Effects

Statins are widely used in clinical practice to treat hyperlipidemia. They act on hepatocytes and inhibit the enzyme, HMG-CoA reductase. This inhibition limits cholesterol biosynthesis and reduces hepatic cholesterol concentrations. Statins can also cause a decrease in plasma triglycerides and a small increase in high-density lipoproteins (HDL). Statins are classified into two groups based on their solubility profile and have slightly different clinical effects. The lipophilic group includes atorvastatin, simvastatin, lovastatin, pitavastatin and fluvastatin. The hydrophilic group includes pravastatin and rosuvastatin. The therapeutic, pharmacokinetic and pharmacodynamic characteristics of common statins are listed in Table 2 (9, 10). At present, the leadership of the National Lipid Association convened an Expert Panel to develop a consensus set of recommendations for patient-centered management of dyslipidemia in clinical medicine(11).

### Pleiotropic Effects

**Figure 1.**
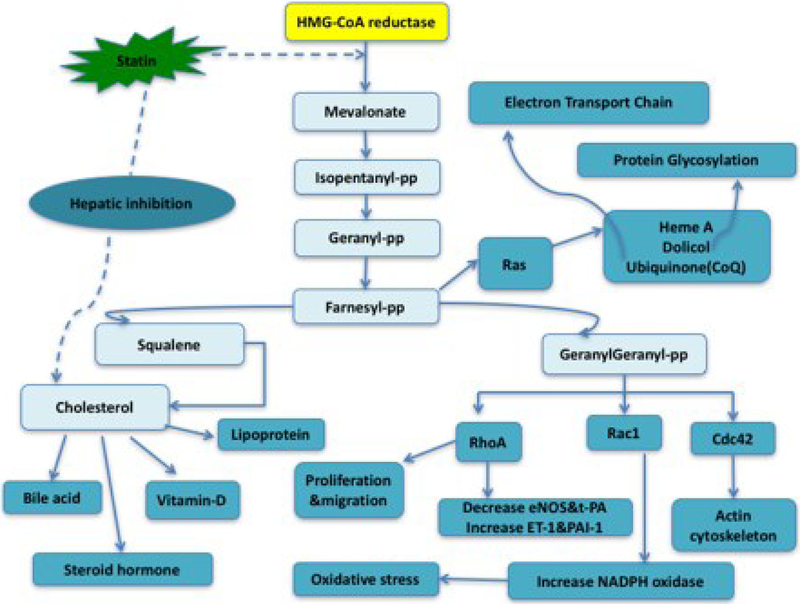
Illustration of the Pleiotropic effect of statins. Cdc42: Cell division control protein 42 homolog, HMG-CoA: 3-Hydroxy-3-methylglutaryl coenzyme A, pp: pyrophosphate, eNOS: endothelial nitric oxide synthase, ET-1: endothelin type 1, NADPH: nicotinamide adenine dinucleotide phosphate, PAI-1: plasminogen activator inhibitor type 1, Rac1: Ras-related C3 botulinum toxin substrate 1, RhoA: Ras homolog gene family, member A, tPA: tissue plasminogen activator. (Part of the figure was adopted from Bedi O, Dhawan V, Sharma PL, Kumar P. Pleiotropic effects of statins: new therapeutic targets in drug design. Naunyn Schmiedebergs Arch Pharmacol 2016; 389: 695-712 published by Springer with permission.)

Current studies have shown that statins may have many additional benefits that are unrelated to the lipid-lowering activity. This lipid-independent effect, the pleiotropic effect, is responsible for the anti-inflammation, anti-oxidative stress, anti-proliferation and anti-thrombosis properties of statins. The pleiotropic effect is also thought to stabilize atherosclerotic plaques and improve endothelial function and immunomodulation. The primary mechanism of the pleiotropic effect may be due to the inhibition of two signaling pathways: Rho/ROCK (rho-associated coiled-coil-containing protein kinase) and Rac (a monomeric G protein and member of the small GTPase subfamily). This inhibition activates peroxisome proliferator-activated receptors (PPARs) which may be responsible for the pleiotropic effect of statins (Figure 1) (10). It has also been suggested that the purinergic signaling pathway may contribute to cardiovascular protection (12).

### Adverse Effects

Although the benefits of statins have been well documented, adverse effects have also been reported. The most common side effects, myotoxicity and statin-associated muscle symptoms (SAMS), often result in poor medication compliance (13). The European Atherosclerosis Society Consensus Statement Panel places the incidence of SAMS between 7%~29% (14). The symptoms of SAMS usually occur within the first 6 months of starting statin therapy and disappear after statin doses are reduced. The most serious adverse event, rhabdomyolysis, occurs very rarely and may lead to AKI, disseminate intravascular coagulation (DIC) and death (15). Liver toxicity characterized by elevated blood concentrations of transaminases is also reported as a potential adverse effect of statin therapy. It has also been suggested that statin therapy may increase the incidence of diabetes (16); however, the mechanism is still unclear. Adverse events may also be increased in the elderly as these patients often require higher doses of statin therapy and have concomitant medications that may have interactions with the drug (11, 17).

## Effects of Statins on Perioperative Complications

### Effect on Atrial Fibrillation

Patients undergoing cardiac surgery are at high risk for developing perioperative arrhythmias. Atrial fibrillation (AF) is the most common arrhythmia after cardiac surgery and occurs in 25%~40% of patients undergoing coronary artery bypass grafting (CABG) or valve surgery. It is associated with increased incidences of stroke, mortality, LOS and cost (18). Changes to the atrial conducting pathway secondary to inflammatory reactions and oxidative stress may be the most important factor leading to the development of postoperative AF (POAF) (19). In one prospective and randomized study, patients were given 40 mg of atorvastatin per day for 30 days after surgery. The results indicated that postoperative statin treatment reduced new-onset POAF and decreased C reaction protein (CRP) levels in the patients undergoing isolated CABG (20). One study of 984 participants undergoing on-pump or off-pump cardiac surgery showed that statin treatment prior to surgery reduced the incidence of POAF (OR 0.40; 95% CI: 0.29 to 0.55; P < 0.01) (21). In a study of 1890 CABG patients, perioperative atorvastatin treatment is not found to be associated with reduced postoperative atrial fibrillation (22). Another study of 11 randomized controlled trials (RCT) that included 1,105 participants underwent cardiac surgery. It excluded patients who had received statin therapy for longer than 3 weeks in order to evaluate the anti-arrhythmic effects of statins due to the early pleiotropic effects rather than the later lipid-lowering effects. The results showed that participants with statin pretreatment had a lower occurrence of POAF (19% vs 36%; OR 0.41; 95% CI 0.34-0.54; P < 0.00001) (23). Although most clinical studies support that perioperative statin reduces the incidence of POAF, some negative results have also been published. In one study, each patient was given either rosuvastatin 20 mg once daily or matching placebo tablets for up to 8 days prior to surgery and 5 days after surgery. While the postoperative concentrations of LDL-C and CRP were lower in patients who received rosuvastatin (P < 0.001), the rate of POAF was not different (P = 0.72) (24). The authors hypothesized that the different type of statins, the use of beta-blockade and a younger patient population with good cardiac function may have contributed to the results.

### Reducing Myocardial Infarction

Perioperative MI is another complication that can increase the morbidity and mortality associated with surgery. Both surgery and anesthesia stimulate catecholamine production, leading to an increase in heart rate, myocardial contractility and oxygen demand. While many patients are able to tolerate these changes, patients with cardiovascular disease are at increased risk for myocardial ischemia and adverse cardiac events. Operative stress and cardiopulmonary bypass (CPB) also trigger a systematic inflammatory response which has been reported to contribute to cardiac events (25).

The pleitropic effect of statins may reduce these events. Martínez-Comendador and colleagues conducted a prospective trial focused on the markers of myocardial damage in 164 patients who had elevated preoperative interleukin-6 (IL-6) levels. The study showed that statin pretreatment was associated with lower postoperative IL-6 and troponin I levels (26). A meta-analysis of 15 RCTs with 2,292 participants suggested that perioperative statin treatment reduced the risk of MI in cardiac and noncardiac surgery (27). Another study of 16 RCT and 2,275 patients revealed a significant reduction in MI in statin-naive high risk patients underwent heart surgery and received statin pretreatment (RR 0.54, 95% CI 0.38–0.76, P < 0.001) (28).

### Reducing Vascular Thrombosis and Improving Graft Patency

Saphenous vein grafts (SVG) have lower long term patency rates when compared to internal mammary artery grafts. The most common mechanisms leading to SVG failure are acute thrombosis and intimal hyperplasia in the first year after surgery, and atherosclerosis in later stages (29). Vessel diameter and surgical technique also contribute. By improving endothelial function, preventing graft spasms, inhibiting platelet activation and thrombus formation, attenuating intimal hyperplasia, reducing vascular inflammation and regulating the redox state, many studies have studied whether statins may have a role in the prevention of SVG thrombosis (30-33). The JUPITER study, a randomized, double-blind, placebo-controlled, multicenter trial conducted at 1,315 sites in 26 countries, found that rosuvastatin significantly reduced the occurrence of symptomatic venous thromboembolism in healthy persons (34). A retrospective study found that patients on statins had higher primary-revised (94% vs 83%; P < 0.02) and secondary (97% vs 87%; P < 0.02) graft patency rates after 2 years. They also found the risk of graft failure was 3.2-fold higher in the control group (Table 1) (35). In another RCT, 42 statin-naïve patients undergoing elective CABG were randomized to receive either atorvastatin 40 mg/d or placebo for 3 days before surgery. The results indicated that even short-term statin treatment during the preoperative period might have a beneficial effect on SVG biology and the redox state (36). One study with a median follow-up of 1.9 years also demonstrated that statin treatment reduced the risk of venous thromboembolic disease by 43% (5). Of note, different types of statins may have different effects on vessels; lipophilic statins such as atorvastatin and simvastatin may have more significant vascular protective effects than hydrophilic statins such as rosuvastatin (37).

### Effect on Acute Kidney Injury

Acute kidney injury (AKI) is another common complication after cardiac surgery and can have an incident rate as high as 33% (38). As it is often accompanied with significantly increased morbidity and mortality, the prevention of AKI is crucial to improving outcomes after cardiac surgery. Potential therapeutic agents often aim to decrease oxidative stress, endothelial dysfunction and vascular inflammation. Statins have many of these properties; however, the literature remains split on the ability of statins to significantly reduce postoperative AKI. One study showed that pitavastatin has eNOS-independent protective actions against angiotensin II-induced renal insufficiency through the inhibition of the transforming growth factor (TGF)-β-Smad 2/3 signaling pathway and the suppression of oxidative stress (39). One study of 99,392 cardiovascular surgery patients from 1995 to 2008, the authors demonstrated statin use was associated with significantly lower rates of AKI (OR, 0.84; 95% CI, 0.78 to 0.90), acute dialysis (OR, 0.83; 95% CI, 0.72 to 0.95) and mortality (OR, 0.79; 95% CI, 0.74 to 0.85) (40). Another study of 59,771 patients also demonstrated that preoperative statin therapy reduced the incidence of postoperative renal dysfunction (OR 0.89, 95% CI 0.84-0.95, P < 0.0001) and the need of postoperative renal replacement therapy (RRT) (OR 0.76, 95% CI 0.62-0.92, P = 0.006) (7).

Other studies did not reach the same conclusion. A study of 7 RCTs with 662 participants showed perioperative statin use was not associated with a decreased incidence of AKI in adults undergoing cardiac surgery with CPB (41). A meta-analysis of 91,419 cardiac surgery patients from 54 trials (12 RCT, 42 observational) compared the incidences of AKI in patients who received perioperative statin therapy to the incidences of AKI in patients who did not receive statin therapy and found no significant difference between the two groups (42). Other studies also failed to show a difference in the incidence of AKI in patients undergoing cardiac surgery with CPB (43-45). One study showed that rosuvastatin pretreatment was actually associated with an increased rate (5.4 ± 1.9) of postoperative AKI (P = 0.005) (24). However, it is still controversial whether statins provide renal protection.

### Effect on Postoperative Infection

Infection after major surgery is always a concern to clinicians. Factors associated with an increased risk of infection after cardiac surgery include diabetes mellitus (DM), heart failure, chronic obstructive pulmonary disease (COPD), increased age, elevated baseline creatinine level and a longer duration of CPB (46). While the anti-inflammatory effects of statins have been well studied, it is unclear if they improve post-surgical infection rates. Statins inhibit the production of several proinflammatory cytokines, including TNF-α, IL-6, IL-8, reduce the CRP level and suppress the proliferation of natural killer cells. Some clinical studies indicate perioperative statin therapy may be associated with lower rates of infection (47-50); however, other studies have failed to find sufficient evidence (51,52). In short, more large-size prospective clinical trials are needed to study the effect of statins on postoperative infection.

### Other Outcomes

Statin therapy may have other benefits in the perioperative time period. One meta-analysis of 19 studies with 31,725 patients found that statin exposure significantly reduced postoperative 30-day mortality (OR 0.57; CI 95% 0.49-0.67) and stroke (OR 0.74; CI 95% 0.60-0.91) (53). Two recent large studies also confirmed that preoperative statin therapy reduced short-term mortality, stroke and LOS (6, 42). The data from Cochrane Collaboration further suggested that preoperative statin therapy shortens the ICU and hospital length of stay (21). Allou and colleagues studied the effects of statin pretreatment on high-risk patients with 2 or more cardiovascular risk factors undergoing isolated cardiac valve surgery. In their multivariate analysis, after adjustment with a propensity score, the results showed statin pretreatment reduced postoperative mortality significantly in these patients (OR=0.41; 95% CI, 0.17-0.97; P = 0.04) (54). Another study showed statin therapy was associated with increased long-term survival post-aortic valve replacement surgery with a biological valve (median follow-up was 5.8 years). Interestingly, there was no benefit in patients undergoing mitral valve repair or a mitral valve replacement with a mechanical valve. This finding may be in part due to the warfarin therapy associated with mechanical valves as warfarin may interfere with the pleiotropic effect. It is also possible that statins have a mechanism of action on the biological tissue of the valve repair or replacement which is unclear (55).

### Statin and New-Onset Diabetes and Insulin Resistance

Many studies have found that statins actually increase the risk of new-onset DM and insulin resistance (56, 57). In a prospective study of 120 non-diabetic patients with dyslipidemia, patients were treated with a lipophilic statin for three months. Insulin sensitivity decreased by 20% in the statin group when compared to the control group and the blood glucose was found to be higher in the statin group in ICU (P < 0.001). Multiple regression analysis studies also found that lipophilic statin use was independently associated with intraoperative insulin sensitivity (P = 0.03) (58). In another study that analyzed 29 trials with 163,039 participants, the authors found that statins significantly increased the likelihood of developing DM by 12%. Atorvastatin was associated with the highest risk of DM (OR 1.34; 95% CI 1.14-1.57) followed by rosuvastatin (OR 1.17; 95% CI 1.02-1.35), simvastatin, atorvastatin, pravastatin, lovastatin and pitavastatin. High-dose atorvastatin increased the odds of developing diabetes even when compared with pravastatin, simvastatin and low-dose atorvastatin (59). While an exact mechanism remains unknown, new-onset DM appears to be related to the dose and potency of the statin. Since the patients involved in these studies were treated with statins for at least 2 months before surgery, studies are needed to evaluate the effects of statins preoperative shot term use and the risk of DM.

### Risk of Perioperative Statin Withdrawal

While statins are generally considered to be safe drugs, perioperative withdrawal of statin therapy has been associated with serious complications. Abrupt statin cessation can reverse the beneficial effects and up-regulate inflammatory molecule production. This can potentially lead to vulnerable plaque rupture, which is thought to be one of the main causative mechanisms of perioperative cardiac complications (60). In a retrospective observational study of 68,606 non–ST-segment elevation MI patients, statin cessation during the first 24 hours of hospitalization was independently associated with adverse outcomes, including in-hospital death, cardiac arrest, and cardiogenic shock (61). In a prospective observational study of 669 patients undergoing infrarenal aortic surgery, the authors concluded that postoperative statin withdrawal (>4 days) was an independent predictor of postoperative myonecrosis (OR 2.9; 95% CI 1.6-5.5) (62). Another study looked at 298 patients who had been on long-term statin therapy and were scheduled for elective major vascular surgery. The results showed that statin discontinuation was associated with an increased risk for postoperative troponin release, myocardial infarction and cardiovascular death. The results also indicated that extended-release fluvastatin was associated with fewer perioperative cardiac events than atorvastatin, simvastatin or pravastatin (60). Unfortunately, as statins are only available in oral formulation, patients undergoing major surgery who are unable to take oral medications, will be at increased risk for these complications.

## Conclusion and the Future

Statins possess many properties that may benefit patients undergoing cardiac surgery. Their ability to lower lipids, improve endothelial function and blood flow, reduce vascular inflammation, stabilize atherosclerotic plaques, inhibit the myocardial redox state and attenuate platelet aggregation and vascular remodeling, make statins ideal drugs to prevent many of the postoperative complications of cardiac surgery. At present, most studies have shown a reduced incidence of major cardiovascular events, including MI, AF, infection, stroke and death. Other studies also suggest that statin treatment may improve graft patency by reducing venous thrombosis after vascular surgery. Length of hospital stay and time spent in the ICU have also been shown to be shorter in patients receiving statin therapy perioperatively. Despite the many positive effects of statin therapy, there are also potential limitations to statin therapy. The effect on kidney function remains controversial and will need further investigations. Statins may also increase the risk of new-onset DM. Rarely, statins can be associated with myotoxicity, SAMS and rhabdomyolysis. The risk of statin withdrawal is also concerning in patients who are unable to take oral statins. Nonetheless, more large-scale clinical trials evaluating the different types of statins, the optimal dosing and duration of therapy are needed to fully evaluate the risks and benefits of statin therapy for cardiac surgery.
