# Supplementary material for: Perioperative statin therapy in cardiac surgery: a meta-analysis of randomized controlled trials
Source: Crit Care. 2016 Dec 5;20:395. doi: 10.1186/s13054-016-1560-6 (PMC5139027; doi:10.1186/s13054-016-1560-6)
Supplement: Supplementary file 1 [file 13054_2016_1560_MOESM1_ESM.pdf]

SUPPLEMENTARY MATERIAL

Perioperative statin therapy in cardiac surgery: a meta-analysis of randomized controlled trials

AUTHORS

Alessandro Putzu, Bruno Capelli, Alessandro Belletti, Enrico Ferrari, Michele Gallo, Gabriele Casso, Tiziano Cassina, Giovanni Landoni

SUMMARY

Supplemental Methods: PRISMA 2009 Checklist 2
Supplemental Methods: Search strategy for PubMed........ 4
Supplemental Methods: Bias Risk Assessment 5
Supplemental Methods: Trial Sequential Analysis........ 6
Supplemental Results: Reasons of Major Exclusion 7
Supplemental Results: Further Characteristics of the included trial........ 8
Supplemental Results: Bias Risk Assessment 10
Supplemental Results: GRADEpro summary of findings table 11
Supplemental Results: Acute Kidney Injury........ 13
Supplemental Results: Atrial Fibrillation 16
Supplemental Results: Myocardial infarction........ 19
Supplemental Results: Stroke 22
Supplemental Results: Infections........ 26
Supplemental Results: Mortality........ 29
Supplemental Results: Clinical outcomes and perioperative statin regimen 31
Supplemental Results: Clinical outcomes in patients on chronic statin therapy or statin-naïve........ 32
Supplemental Results: Clinical outcomes and CABG surgery........ 33
Supplemental Results: Influence of publication year and trial size on clinical outcomes. 34
Supplemental Results: Higher-risk of bias trials and clinical outcomes........ 35
Supplemental references 36

Supplemental Methods: PRISMA 2009 Checklist

Section/topic	#	Checklist item	Reported on page #
**TITLE**			
Title	1	Identify the report as a systematic review, meta-analysis, or both.	1
**ABSTRACT**			
Structured summary	2	Provide a structured summary including, as applicable: background; objectives; data sources; study eligibility criteria, participants, and interventions; study appraisal and synthesis methods; results; limitations; conclusions and implications of key findings; systematic review registration number.	3, 4
**INTRODUCTION**			
Rationale	3	Describe the rationale for the review in the context of what is already known.	5
Objectives	4	Provide an explicit statement of questions being addressed with reference to participants, interventions, comparisons, outcomes, and study design (PICOS).	5
**METHODS**			
Protocol and registration	5	Indicate if a review protocol exists, if and where it can be accessed (e.g., Web address), and, if available, provide registration information including registration number.	6
Eligibility criteria	6	Specify study characteristics (e.g., PICOS, length of follow-up) and report characteristics (e.g., years considered, language, publication status) used as criteria for eligibility, giving rationale.	6
Information sources	7	Describe all information sources (e.g., databases with dates of coverage, contact with study authors to identify additional studies) in the search and date last searched.	6
Search	8	Present full electronic search strategy for at least one database, including any limits used, such that it could be repeated.	6, Supplemental
Study selection	9	State the process for selecting studies (i.e., screening, eligibility, included in systematic review, and, if applicable, included in the meta-analysis).	6, 7
Data collection process	10	Describe method of data extraction from reports (e.g., piloted forms, independently, in duplicate) and any processes for obtaining and confirming data from investigators.	7
Data items	11	List and define all variables for which data were sought (e.g., PICOS, funding sources) and any assumptions and simplifications made.	6, 7

Risk of bias in individual studies	12	Describe methods used for assessing risk of bias of individual studies (including specification of whether this was done at the study or outcome level), and how this information is to be used in any data synthesis.	7
Summary measures	13	State the principal summary measures (e.g., risk ratio, difference in means).	7
Synthesis of results	14	Describe the methods of handling data and combining results of studies, if done, including measures of consistency (e.g., I^2) for each meta-analysis.	7, 8
Risk of bias across studies	15	Specify any assessment of risk of bias that may affect the cumulative evidence (e.g., publication bias, selective reporting within studies).	8, 9
Additional analyses	16	Describe methods of additional analyses (e.g., sensitivity or subgroup analyses, meta-regression), if done, indicating which were pre-specified.	7, 8, 9
**RESULTS**			
Study selection	17	Give numbers of studies screened, assessed for eligibility, and included in the review, with reasons for exclusions at each stage, ideally with a flow diagram.	10, Supplemental
Study characteristics	18	For each study, present characteristics for which data were extracted (e.g., study size, PICOS, follow-up period) and provide the citations.	10, Table, Supplemental
Risk of bias within studies	19	Present data on risk of bias of each study and, if available, any outcome level assessment (see item 12).	10, Supplemental
Results of individual studies	20	For all outcomes considered (benefits or harms), present, for each study: (a) simple summary data for each intervention group (b) effect estimates and confidence intervals, ideally with a forest plot.	11,12, Supplemental
Synthesis of results	21	Present results of each meta-analysis done, including confidence intervals and measures of consistency.	11, 12, Suppl.
Risk of bias across studies	22	Present results of any assessment of risk of bias across studies (see Item 15).	10, Suppl.
Additional analysis	23	Give results of additional analyses, if done (e.g., sensitivity or subgroup analyses, meta-regression [see Item 16]).	11-14, Suppl.
**DISCUSSION**			
Summary of evidence	24	Summarize the main findings including the strength of evidence for each main outcome; consider their relevance to key groups (e.g., healthcare providers, users, and policy makers).	15
Limitations	25	Discuss limitations at study and outcome level (e.g., risk of bias), and at review-level (e.g., incomplete retrieval of identified research, reporting bias).	15-19
Conclusions	26	Provide a general interpretation of the results in the context of other evidence, and implications for future research.	20
**FUNDING**			
Funding	27	Describe sources of funding for the systematic review and other support (e.g., supply of data); role of funders for the systematic review.	6

From: Moher D, Liberati A, Tetzlaff J, Altman DG, The PRISMA Group (2009). Preferred Reporting Items for Systematic Reviews and Meta-Analyses: The PRISMA Statement. PLoS Med 6(7): e1000097. doi:10.1371/journal.pmed1000097

For more information, visit: **www.prisma-statement.org**.

Supplemental Methods: Search strategy for PubMed

(((statin*[tiab] OR ((“hydroxymethylglutaryl-CoA reductase”[tiab] OR "HMGCoA reductase"[tiab]) AND inhibitor*[tiab]) OR anticholesteremic[tiab] OR simvastatin[tiab] OR rosuvastatin*[tiab] OR pravastatin*[tiab] OR atorvastatin*[tiab] OR fluvastatin*[tiab] OR cerivastatin*[tiab] OR pitavastatin*[tiab] OR lovastatin*[tiab]) AND (cardiac surgery OR cardiovascular surgery OR heart surgery OR coronary artery bypass graft OR valve replacement OR valve repair OR coronary surgery)) AND (randomized controlled trial[pt] OR controlled clinical trial[pt] OR randomized controlled trials[mh] OR random allocation[mh] OR double-blind method[mh] OR single-blind method[mh] OR clinical trial[pt] OR clinical trials[mh] OR (clinical trial[tw] OR ((singl*[tw] OR doubl*[tw] OR trebl*[tw] OR tripl*[tw]) AND (mask*[tw] OR blind[tw])) OR (latin square[tw]) OR placebos[mh] OR placebo*[tw] OR random*[tw] OR research design[mh:noexp] OR follow-up studies[mh] OR prospective studies[mh] OR cross-over studies[mh] OR control*[tw] OR prospectiv*[tw] OR volunteer*[tw]))) NOT ((animal[mh] NOT human[mh]))

Supplemental Methods: Bias Risk Assessment

We used the Cochrane risk of bias tool [1,2] to evaluate this risk of methodological quality of each included trials. Selection bias (random sequence generation and allocation concealment), performance bias (blinding of participants and investigators), detection bias (blinding of outcomes assessors), attrition bias (incomplete outcome data), reporting bias (selective outcome reporting), and other bias were judged to be of either low, unclear or high risk. The other bias domain included the classic items reported by the "Cochrane Handbook for Systematic Reviews of Interventions" [1] but also the presence of: intention-to-treat analysis, sample size calculation, and ethical approval of the trial.

Supplemental Methods: Trial Sequential Analysis

Trial sequential analysis (TSA) is a methodology that combines an information size calculation for a meta-analysis with the threshold for a statistically significant treatment effect, and, eventually, the threshold for futility or harm of the treatment [3–5]. In particular, TSA apply trial sequential monitoring boundaries applied to meta-analysis, in order to adjust the confidence intervals and decrease type I errors [3–5]. The underlying hypothesis for TSA is that significance testing and calculation of the 95% CIs are performed each time a new study is published and TSA depends on the quantification of the required information size (size of randomized patients).

We performed a post-hoc TSA according to the primary analysis, with intent to maintain an overall 5% risk for type I error and a risk for type II error of 20%, at a power of 80%. We derived the required information size using the proportional event in the control group of the present meta-analysis and we calculated relative risk reduction (RRR) or relative risk increase (RRI) according to the low risk of bias randomized literature and in order to attempt to evidence clinically meaningful differences. The resulting required information size was further diversity (D^2)-adjusted, since I^2 may underestimate the required information size [6]; in case of $D^2 = 0$ we performed a sensitivity analysis assuming a $D^2 = 25\%$.

Supplemental Results: Reasons of Major Exclusion

Nineteen trials were excluded because of:

- 2 manuscripts with overlapping populations [7,8]
- 2 manuscripts with lack of a randomized design [9,10]
- 4 manuscripts with lack of an adequate control group [11–14]
- 1 manuscript with an inappropriate clinical setting [15]
- 10 manuscripts reporting other endpoints [16–25]

Supplemental Results: Further Characteristics of the included trial

Trial	Country	Endpoint of the study	Patients with atrial fibrillation excluded?	Atrial fibrillation assessed by continuous ECG monitoring?	Patients with chronic kidney disease excluded?	Intention-to-treat analysis	Follow-up for mortality	AKI definition
Almansob 2012	China	Myocardial damage	No	No	Yes	No	NA	NA
Baran 2011	Turkey	Endothelial progenitor cells	Yes	NR	Yes	No	Hospital mortality	Renal failure needing dialysis
Berkan 2008	Turkey	P-selectin	NR	NA	Yes	Yes	Hospital mortality	NA
Billings 2016	USA	Acute kidney injury	No	NR	Yes, if end-stage in dialysis	Yes	Hospital mortality	AKIN criteria
Caorsi 2008	Chile	Cytokines	NR	NR	Yes	Yes	Hospital stay	NA
Carrascal 2016	Spain	Atrial fibrillation	Yes	Yes	Yes, if creatinine > 2 mg/dL	Unclear	NA	Renal failure needing dialysis
Castaño 2015	Spain	Myocardial damage and inflammation	NR	NR	No	Yes	30-days	NA
Chello 2006	Italy	Cytokines	No	NR	Yes	Yes	30-days	NR
Christenson 1998	Switzerland	Thrombocytosis and thrombotic complications	NR	NA	NR	Unclear	Hospital mortality	NA
Dehghani 2014	Iran	Atrial fibrillation	Yes	Yes	Yes	Yes	Hospital mortality	NA
Ji 2009	China	Atrial fibrillation	Yes	Yes	Yes	No	NR	NA
Mannacio 2008	Italy	Myocardial damage	No	No	Yes, if creatinine > 2 mg/dL	Unclear	NR	Serum creatinine > 2.5 mg/dL
Melina 2009*	Italy	Atrial fibrillation	NR	NR	NR	Unclear	NA	NA
Park 2016	Korea	Acute kidney injury	No	NR	Yes, if GFR < 15	Yes	Hospital mortality	AKIN criteria
Patti 2006	Italy	Atrial fibrillation	Yes	Yes	Yes, if creatinine > 3 mg/dL	Yes	30-days	NA
Prowle 2012	Australia	Creatinine	No	NR	Yes, if end-stage	Yes	Hospital mortality	RIFLE consensus guidelines
Song 2008	Korea	Atrial fibrillation	Yes	Yes	Yes, if creatinine >2 mg/dL	Yes	Hospital mortality	NA
Spadaccio 2010	Italy	Endothelial progenitor cells and cytokines	NR	NR	Yes	Yes	30-days	NR

Sun 2011	China	Atrial fibrillation	Yes	Yes	Yes	No	NA	NA
Tamayo 2009	Spain	Cytokines	No	NR	Yes	Yes	NR	NA
Vukovic 2010	Serbia	Cardiac index	No	NR	Yes, if end stage CKD in dialysis	Low	NR	NA
Youn 2011	Korea	Major adverse cardiac events	NR	NR	Yes, if creatinine >3 mg/dL	Yes	30-days	NA
Zheng 2016	China	Atrial fibrillation and myocardial injury	Yes	Yes	Yes, if creatinine > 2.3 mg/dL	Low	Hospital mortality	AKIN criteria

AKIN criteria, Acute Kidney Injury Network criteria [26]; RIFLE, Risk, Injury, Failure, Loss of kidney function, and End-stage kidney disease [27]; GFR, glomerular filtration rate; NR, not reported; NA, not applicable; *Abstract-only publication.

Supplemental Results: Bias Risk Assessment

Random sequence generation was assessed as low-risk of bias in 13 trials (57%), allocation concealment in 8 trials (35%), blinding of participants in 12 trials (52%), blinding of outcome assessors in 6 trials (26%), completeness of outcome data in 17 trials (74%), absence of selective outcome reporting in 17 trials (74%), and absence of other bias in 10 trials (43%).

Finally, 3 trials scored low-risk of bias in all bias domains. Five trials scored unclear-risk of bias and 15 trials high-risk of bias.

eFigure 1 - Risk of bias graph: review authors' judgments about each risk of bias item presented as percentages across all included studies.

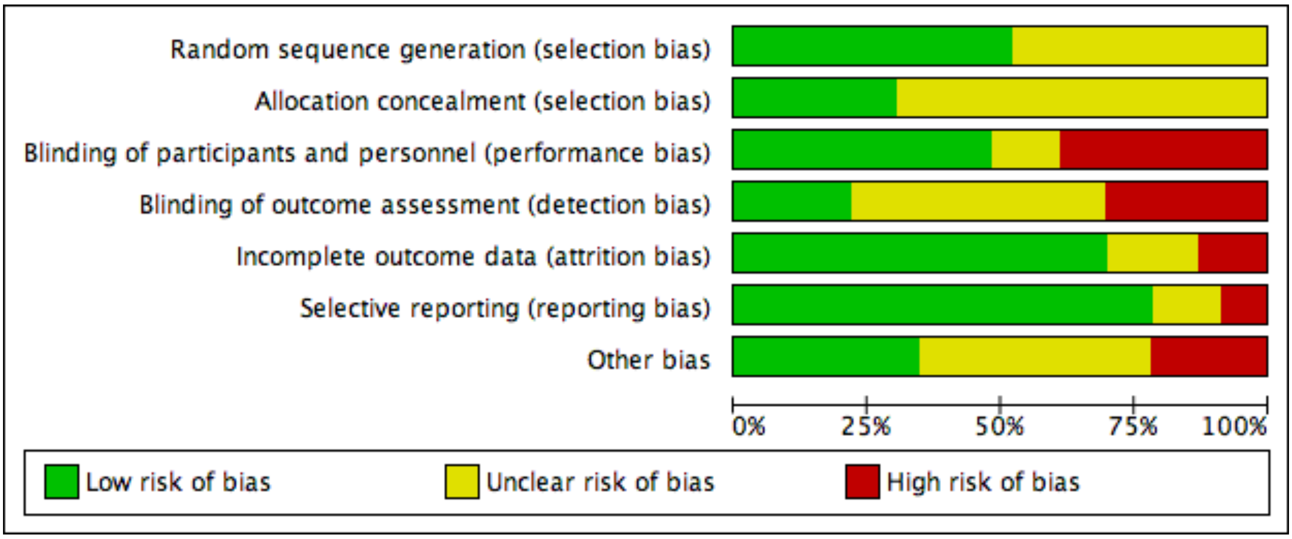

Supplemental Results: GRADEpro summary of findings table

**Quality assessment**							**№ of patients**		**Effect**		**Quality**	
**№ of studies**	**Study design**	**Risk of bias**	**Inconsistency**	**Indirectness**	**Imprecision**	**Other considerations**	**Final**		**Relative (95% CI)**	**Absolute (95% CI)**		
Acute Kidney Injury - Acute kidney injury: low-risk of bias trials												
3	randomised trials	not serious	not serious	not serious	not serious	none	314/1318 (23.8%)	262/1319 (19.9%)	**OR 1.26** (1.05 to 1.52)	**39 more per 1000** (from 8 more to 75 more)	⊕⊕⊕⊕ HIGH	
Atrial fibrillation - Atrial fibrillation: low-risk of bias trials												
2	randomised trials	not serious	not serious	serious [1]	not serious	none	318/1268 (25.1%)	300/1269 (23.6%)	**OR 1.08** (0.90 to 1.30)	**14 more per 1000** (from 18 fewer to 51 more)	⊕⊕⊕◯ MODERATE	
Myocardial infarction - Myocardial infarction: low-risk of bias trials												
2	randomised trials	not serious	not serious	serious [2]	not serious	none	86/1268 (6.8%)	88/1269 (6.9%)	**OR 0.97** (0.71 to 1.33)	**2 fewer per 1000** (from 19 fewer to 21 more)	⊕⊕⊕□ MODERATE	
Stroke - Stroke: low-risk of bias trials												
2	randomised trials	not serious	not serious	serious [3]	serious [5]	none	15/1268 (1.2%)	12/1269 (0.9%)	**OR 1.25** (0.58 to 2.70)	**2 more per 1000** (from 4 fewer to 16 more)	⊕⊕□□ LOW	
Infections - Infections: low-risk of bias trials												
2	randomised trials	not serious	not serious	serious [4]	serious [6]	none	88/1268 (6.9%)	108/1269 (8.5%)	**OR 0.80** (0.60 to 1.07)	**16 fewer per 1000** (from 5 more to 32 fewer)	⊕⊕□□ LOW	

Quality assessment							№ of patients		Effect		Quality	
**№ of studies**	**Study design**	**Risk of bias**	**Inconsistency**	**Indirectness**	**Imprecision**	**Other considerations**	**Final**		**Relative (95% CI)**	**Absolute (95% CI)**		
Mortality - Mortality: low-risk of bias trials												
3	randomised trials	not serious	not serious	not serious	very serious [5,6]	none	9/1318 (0.7%)	2/1319 (0.2%)	**OR 3.84** (0.95 to 15.55)	**4 more per 1000** (from 0 fewer to 22 more)	⊕⊕◯◯ LOW	

CI: Confidence interval; **OR:** Odds ratio; 1, different diagnostic techniques between trials; 2, different clinical definitions of myocardial infarction between trials; 3, different clinical definitions of stroke between trials; 4, substantial different clinical definitions of infection between trials; 5, wide confidence intervals; 6, sparse data.

Supplemental Results: Acute Kidney Injury

The administration of perioperative statins is associated with an increase of postoperative AKI incidence versus placebo in low risk of bias trials (314 of 1318 [23.82%] patients in statin group versus 262 of 1319 [19.86%] patients in the placebo-control group; OR 1.26 [95% CI, 1.05-1.52]; *p* 0.01; NNH 25) (Fig.3), results confirmed by the TSA, that showed firm evidence for a 25% relative risk increase (RRI) (*below*). The overall quality of evidence was high according to GRADE. When including all the eligible trials despite risk of bias, statins were associated with no difference in postoperative AKI (OR 1.18 [95% CI, 0.99-1.41]; *p* 0.06), but possible small studies publication bias was present (*below*). The sensitivity analyses, excluding trials with possible or unclear industrial conflicts of interest or including only placebo-controlled trials or including trials enrolling > 200 patients, showed that statin therapy is associated with increased postoperative AKI versus control among trials with high- to low- risk of bias (respectively: OR 1.23 [1.03, 1.46], *p* 0.02; OR 1.20 [95% CI, 1.01, 1.43], *p* 0.04; OR 1.29 [1.07, 1.57], p 0.08; with no significant heterogeneity). Higher-risk of bias trials showed no difference between statins and control (Additional File 17).

eFigure 2 – Funnel plot of low- to high- risk of bias trials for acute kidney injury, showing asymmetry and suggesting possible publication bias.

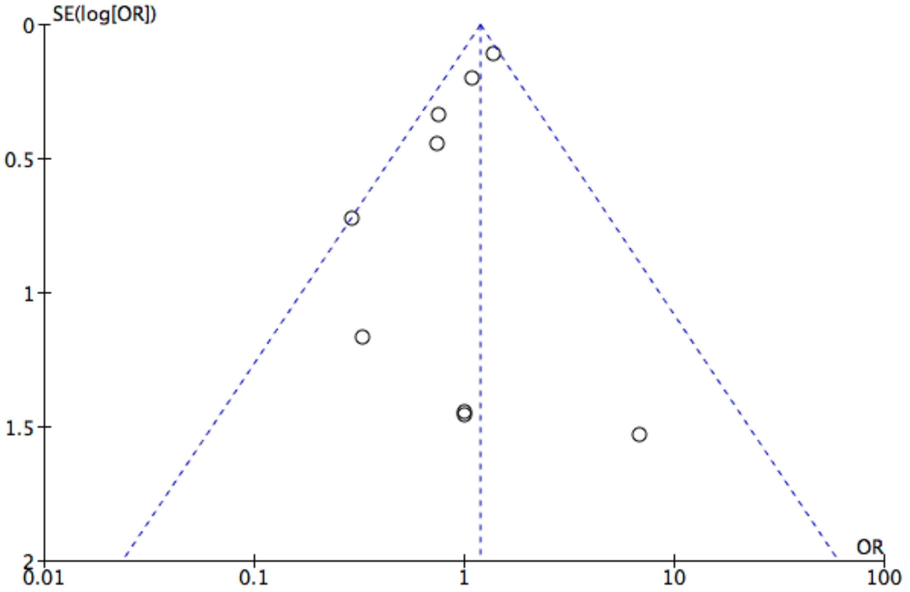

The trial sequential analysis (TSA) for primary analysis was conclusive, since the cumulative z curves crossed the study sequential monitoring boundary of harm (*below*). The overall risk in the control group was 19.86% and a RRI of 25% was assumed based on the current literature [28] and low bias based. The required diversity-adjusted information size is 3085 patients.

eFigure 3 – Trial Sequential Analysis: acute kidney injury in low-risk of bias trials (α = 5%, β = 80%, D^2 = 40.84, RRR = 25%)

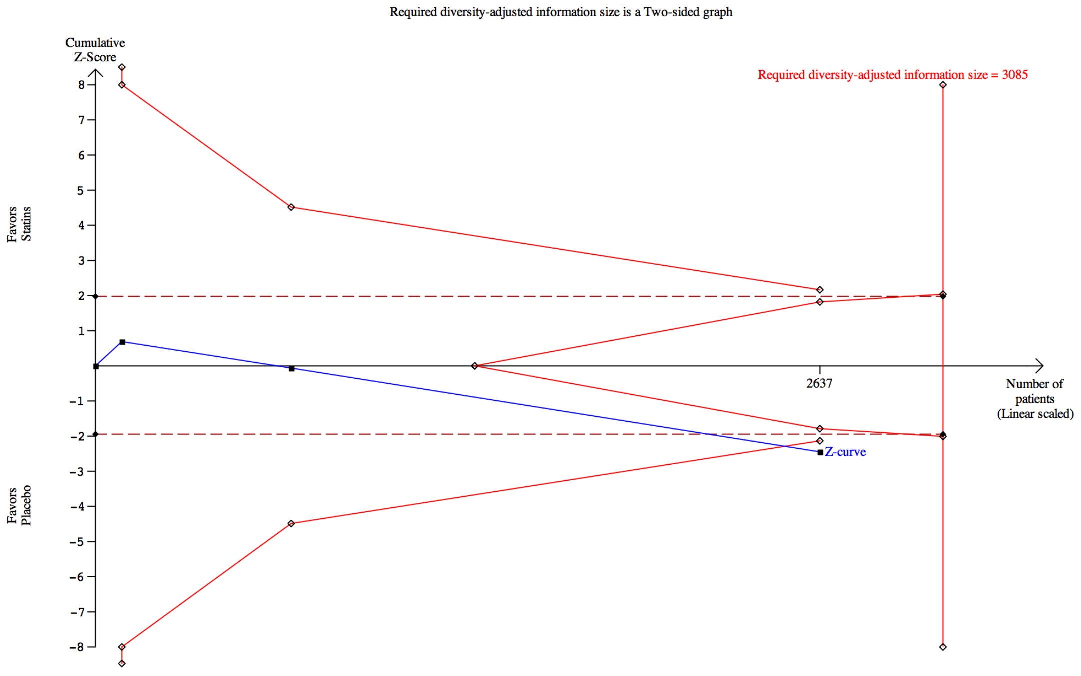

The TSA including all trials despite risk of bias was inconclusive (*below*).

eFigure 4 – Trial Sequential Analysis: acute kidney injury in all the trials (α = 5%, β = 80%, D^2 = 58.34, RRR = 25%)

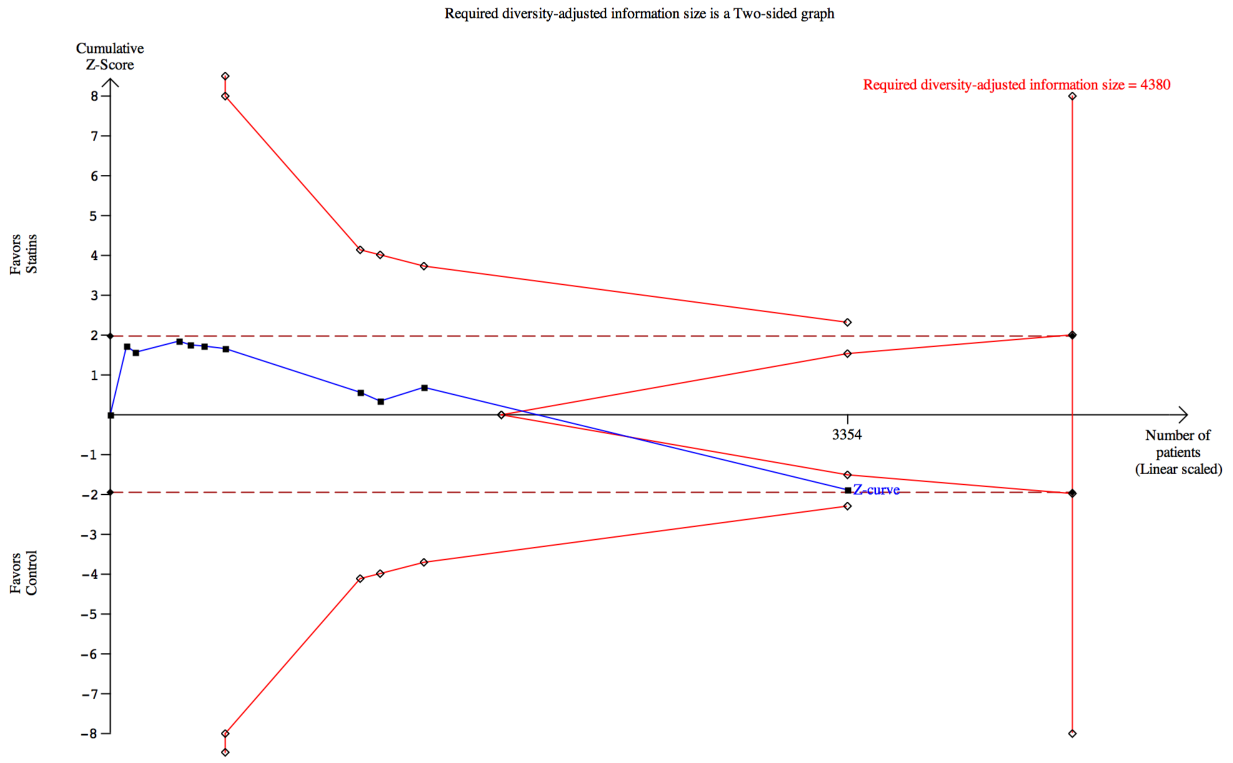

Supplemental Results: Atrial Fibrillation

No difference was found in the rate of postoperative AF in low risk of bias trials (318 of 1268 [25.07%] in the statin group and 300 of 1269 [23.64%] in the placebo group, OR 1.08 [95% CI, 0.90-1.30]; *p* 0.40) (Fig. 4) and the TSA showed futility of the statin treatment when assuming RRR of 20% (*below*). The overall quality of evidence was moderate.

The meta-analysis including all trials despite risk of bias showed a lower incidence of AF among patients allocated to statins, but TSA did not confirm the findings and significant high heterogeneity (p for heterogeneity < 0.0001, I^2 66%) and important small publication bias were present (*below*). Again, sensitivity analyses of the latter results found no significant difference in atrial fibrillation when removing trials with possible or unclear conflicts of interests or when including only trials enrolling more than 200 patients.

eFigure 5 – Funnel plot of low- to high- risk of bias trials for atrial fibrillation, showing important asymmetry and suggesting significant publication bias.

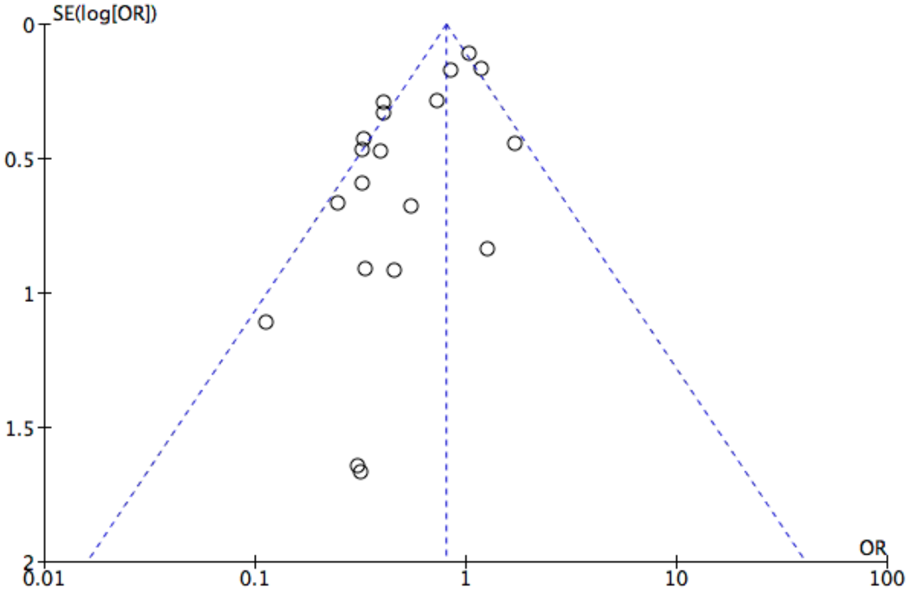

The trial sequential analysis (TSA) for primary analysis was conclusive for futility of statin treatment. The overall risk in the control group was 17.35% and a relative risk reduction of 20% was assumed based on the current literature [29]. The required diversity-adjusted information size is 4027.

eFigure 6 – Trial Sequential Analysis: Atrial Fibrillation in low-risk of bias trials (α = 5%, β = 80%, D^2 = 0, RRR = 20%)

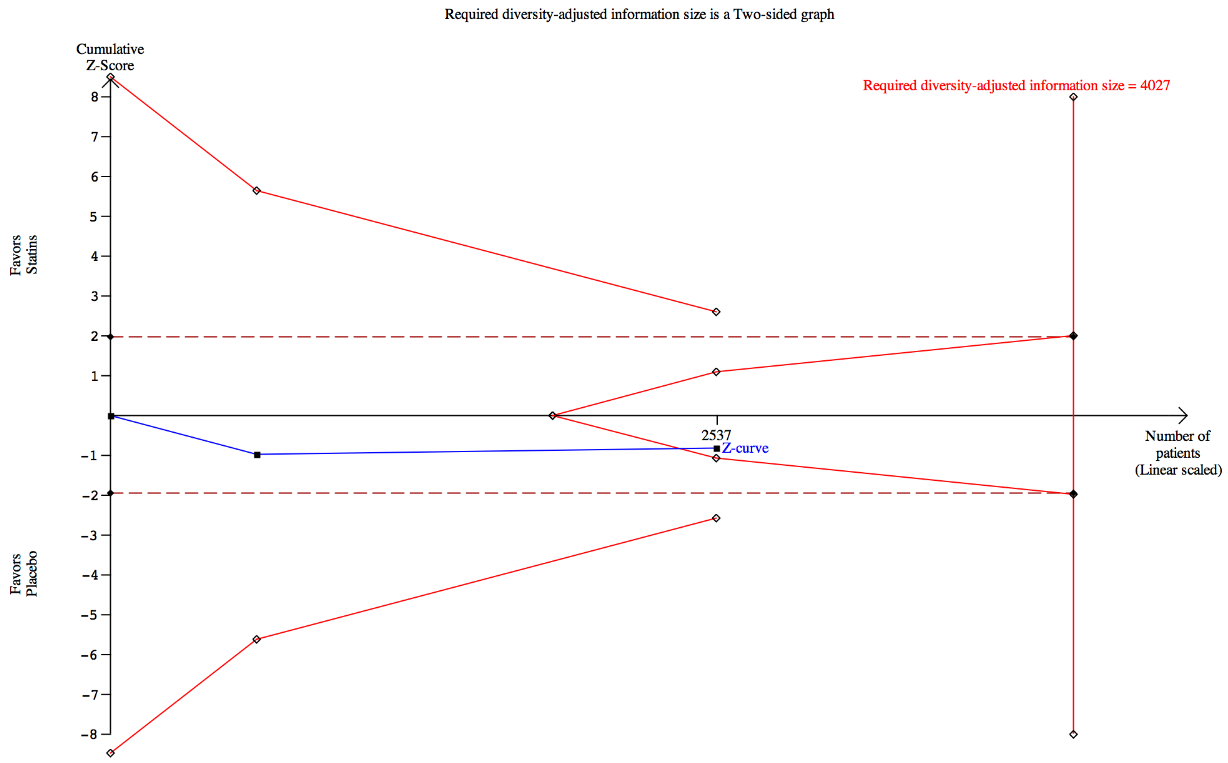

When including trials with higher risk of bias, the TSA was inconclusive.

eFigure 7 – Trial Sequential Analysis: Atrial Fibrillation in all trials (α = 5%, β = 80%, D^2 = 77.18, RRR = 20%)

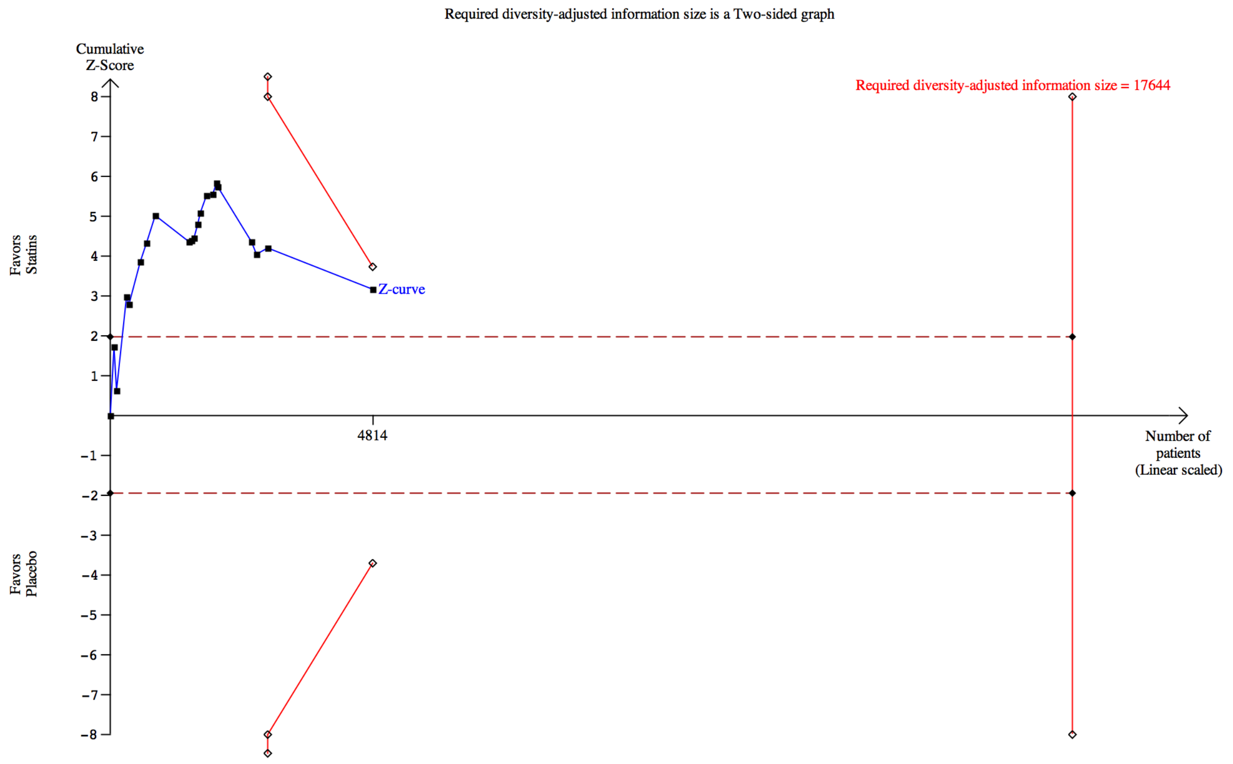

Supplemental Results: Myocardial infarction

The rate of postoperative myocardial infarction did not change significantly between groups in low-risk of bias trials (37 of 960 [3.85%] in statin versus 41 of 962 [4.26%] in the control group; OR 0.90 [95% CI, 0.57-1.42]) (Fig.5) and TSA showed futility of the statin treatment when assuming RRR of 30% (*below*). The overall quality of evidence was moderate.

When including all the trials regardless the risk of bias the results were confirmed (Fig.5), but possible publication bias was present (*below*).

eFigure 8 – Funnel plot of low- to high- risk of bias trials for myocardial infarction, showing asymmetry and suggesting possible small studies publication bias.

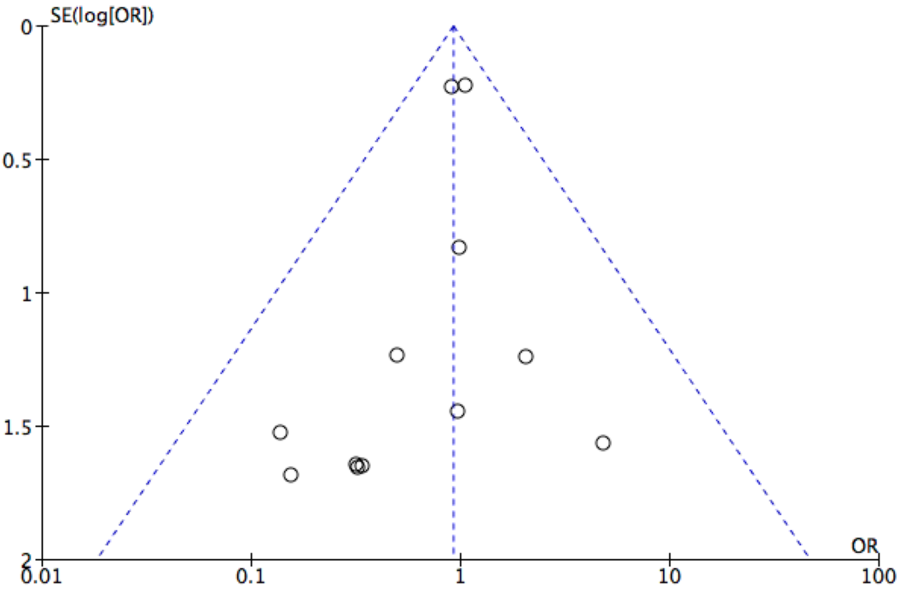

The trial sequential analysis (TSA) was conclusive and demonstrates futility of the treatment, since the cumulative z curves crossed the study sequential monitoring boundary of futility. The relative risk reduction of 30% was assumed based on the clinically meaningful differences between the two treatments. The required information size is 4023.

eFigure 9 – Trial Sequential Analysis: myocardial infarction in low-risk of bias trials (α = 5%, β = 80%, D^2 = 0%, RRR = 30%)

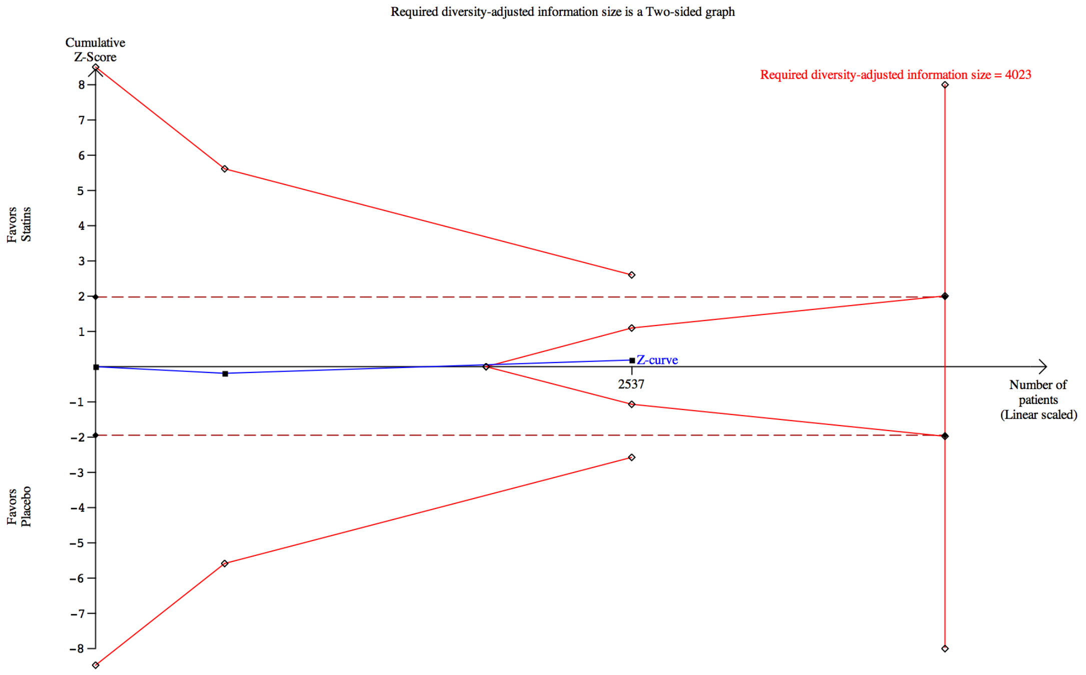

The TSA was conclusive also when including higher risk of bias trials.

eFigure 10 – Trial Sequential Analysis: myocardial infarction in all trials ($\alpha = 5\%$, $\beta = 80\%$, $D^2 = 0$, RRR = 30%)

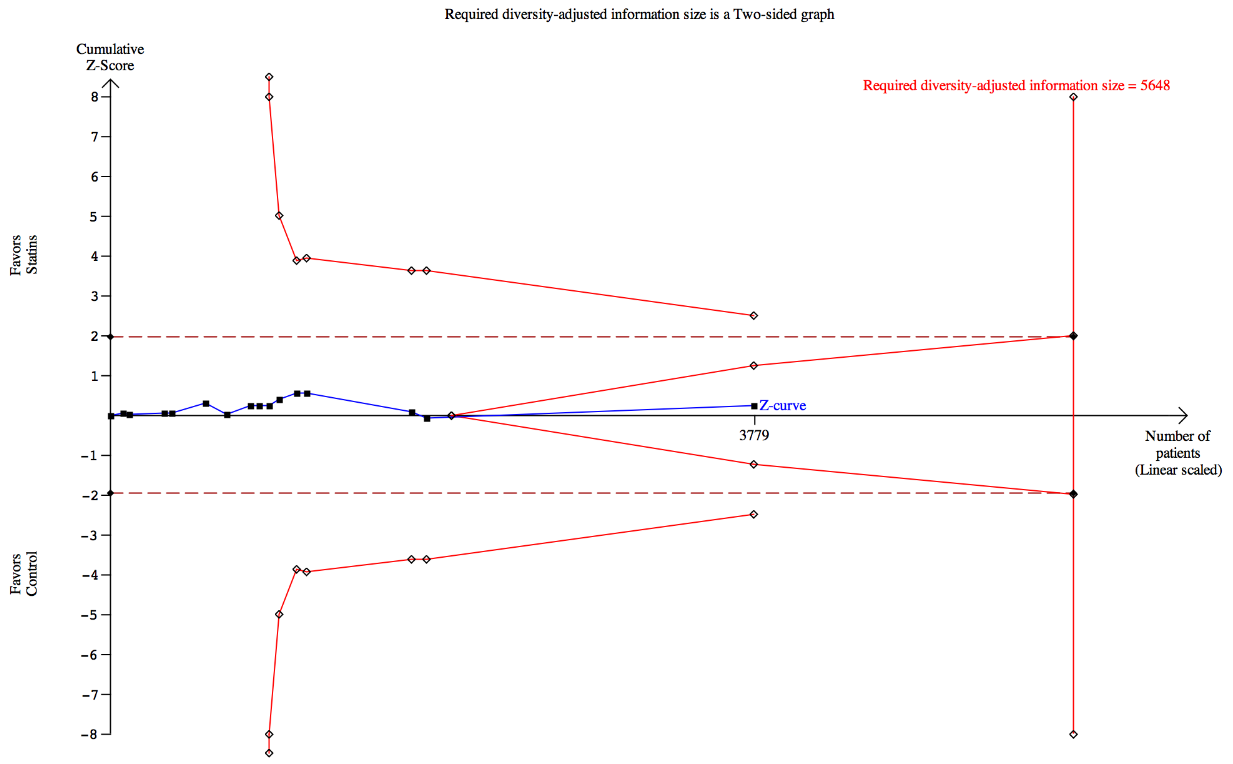

Supplemental Results: **Stroke**

No difference was found in postoperative stroke rate in low-risk of bias trials (15 of 1268 [1.18%] in statin versus 12 of 1269 [0.95%] in the control group; OR 1.25 [95% CI, 0.58-2.70]) (*below*) and TSA showed futility of the statin treatment when assuming RRR of 80% (*below*). The overall quality of evidence was low. Including high- risk of bias trials the results did not change (*below*) and the funnel plot did not show evidence of publication bias (*below*).

eFigure 11 – Forest plot for postoperative stroke.

	Statins		Control			Odds Ratio
**Study or Subgroup**	**Events**	**Total**	**Events**	**Total**	**Weight**	**M-H, Fixed, 95% CI**
**1.4.1 Stroke: low risk of bias trials**						
Billings 2016	10	308	7	307	57.7%	1.44 [0.54, 3.83]
Zheng 2016	5	960	5	962	42.3%	1.00 [0.29, 3.47]
**Subtotal (95% CI)**		**1268**		**1269**	**100.0%**	**1.25 [0.58, 2.70]**
Total events	15		12			
Heterogeneity: $Chi^2 = 0.20$, df = 1 (P = 0.65); $I^2 = 0\%$						
Test for overall effect: Z = 0.58 (P = 0.56)						
**1.4.2 Stroke: all trials**						
Almansob 2012	0	68	0	64		Not estimable
Baran 2012	0	30	0	30		Not estimable
Billings 2016	10	308	7	307	33.8%	1.44 [0.54, 3.83]
Carrascal 2016	1	47	1	43	5.1%	0.91 [0.06, 15.06]
Chello 2006	0	20	0	20		Not estimable
Dehghani 2015	0	29	0	29		Not estimable
Ji 2009	0	71	1	69	7.5%	0.32 [0.01, 7.97]
Mannacio 2008	0	100	0	100		Not estimable
Park 2016	4	100	4	100	19.1%	1.00 [0.24, 4.11]
Song 2008	2	62	2	62	9.6%	1.00 [0.14, 7.33]
Spadaccio 2010	0	25	0	25		Not estimable
Zheng 2016	5	960	5	962	24.8%	1.00 [0.29, 3.47]
**Subtotal (95% CI)**		**1820**		**1811**	**100.0%**	**1.09 [0.60, 2.00]**
Total events	22		20			
Heterogeneity: $Chi^2 = 0.92$, df = 5 (P = 0.97); $I^2 = 0\%$						
Test for overall effect: Z = 0.29 (P = 0.77)						
Test for subgroup differences: $Chi^2 = 0.08$, df = 1 (P = 0.78), $I^2 = 0\%$						

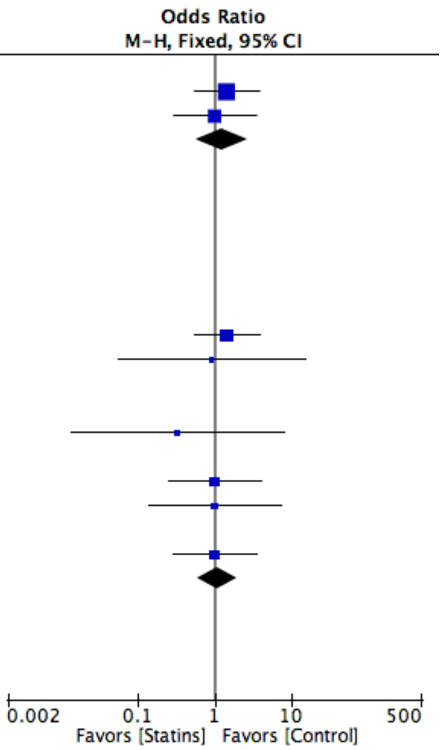

eFigure 12 – Funnel plot of low- to high- risk of bias trials for myocardial infarction.

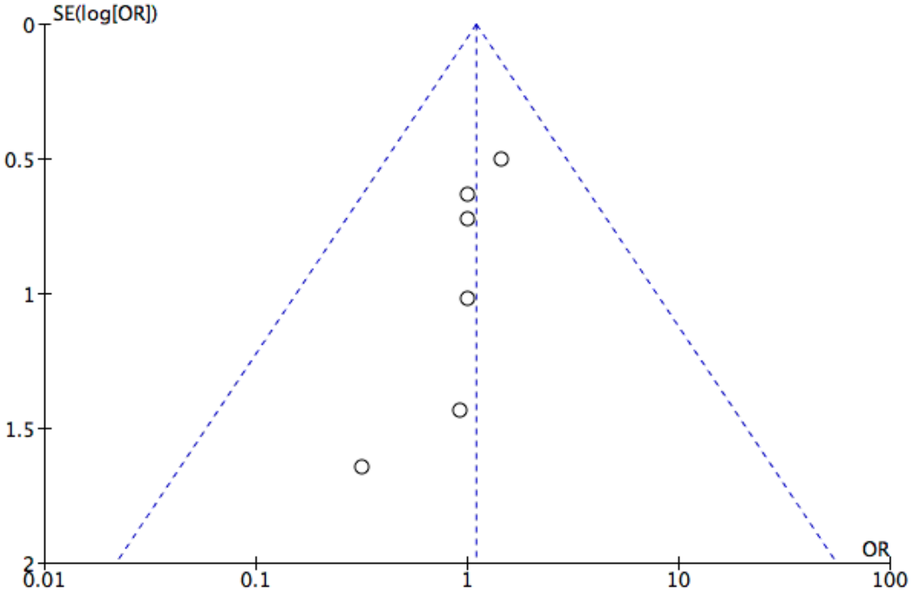

The trial sequential analysis (TSA) for primary analysis was conclusive and demonstrates futility of the treatment, since the cumulative z curves crossed the study sequential monitoring boundary of futility. The relative risk reduction of 80% was assumed based on the clinically meaningful differences between the two treatments, also in consideration of the very low incidence of the event (1%). The required information size is 3081 patients.

eFigure 13 – Trial Sequential Analysis: stroke in low-risk of bias trials ($\alpha = 5\%$, $\beta = 80\%$, D2 = 0%, RRR = 80%)

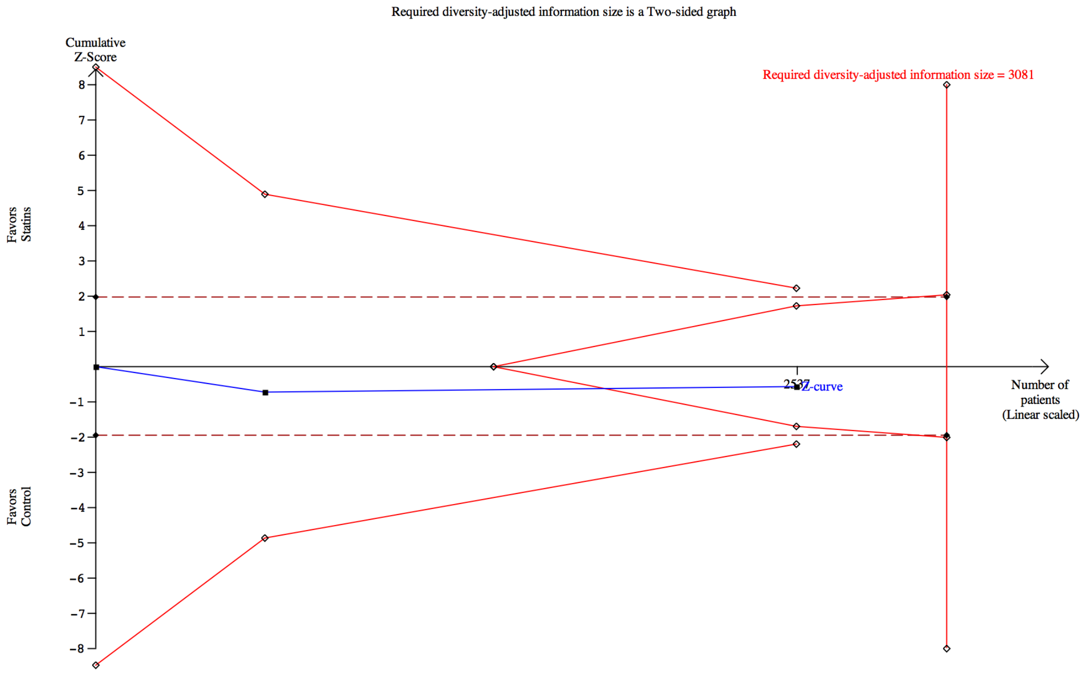

The TSA was conclusive when including all eligible trials.

eFigure 14 – Trial Sequential Analysis: stroke in all the trials despite risk of bias (α = 5%, β = 80%, D2 = 0%, RRR = 80%)

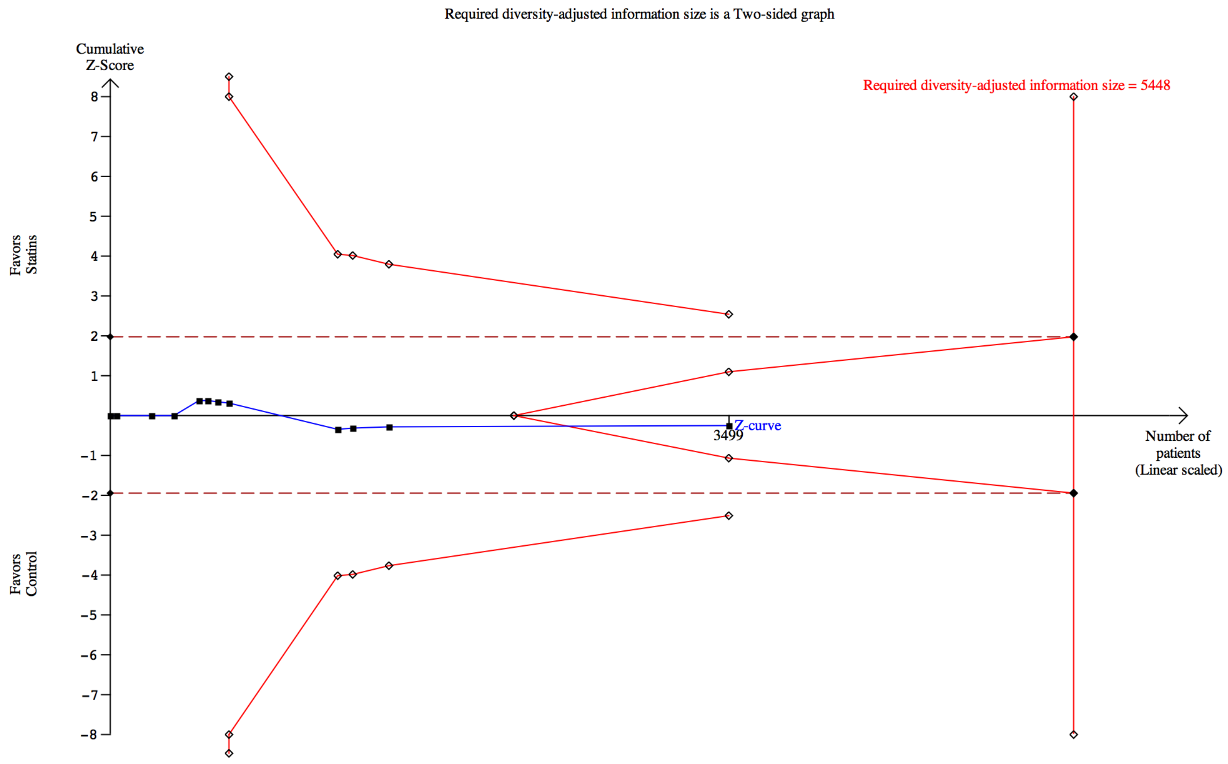

Supplemental Results: Infections

No difference in postoperative infection rate was found between statin and control groups in low-risk of bias trials (88 of 1268 [6.94%] patients in statin group versus 108 of 1269 [8.51%] patients in the control group; OR 0.80 [95% CI, 0.60, 1.07]) (*below*), also when including higher-risk of bias trials (*below*). TSA showed futility of the statin treatment only when including all the trials despite the risk of bias (*below*). The overall quality of evidence was low.

eFigure 15 – Forest plot for postoperative infections.

Study or Subgroup	Statins Events	Statins Total	Control Events	Control Total	Weight	Odds Ratio M-H, Fixed, 95% CI
**1.5.1 Infection: low-risk of bias**						
Billings 2016	8	308	18	307	17.6%	0.43 [0.18, 1.00]
Zheng 2016	80	960	90	962	82.4%	0.88 [0.64, 1.21]
**Subtotal (95% CI)**		**1268**		**1269**	**100.0%**	**0.80 [0.60, 1.07]**
Total events	88		108			
Heterogeneity: $Chi^2 = 2.44$, df = 1 (P = 0.12); $I^2 = 59\%$						
Test for overall effect: Z = 1.48 (P = 0.14)						
**1.5.2 Infection: all trials**						
Billings 2016	8	308	18	307	16.7%	0.43 [0.18, 1.00]
Chello 2006	0	20	1	20	1.4%	0.32 [0.01, 8.26]
Christenson 1999	0	40	37	0		Not estimable
Spadaccio 2010	0	25	1	25	1.4%	0.32 [0.01, 8.25]
Vukovic 2011	1	29	2	28	1.9%	0.46 [0.04, 5.43]
Zheng 2016	80	960	90	962	78.6%	0.88 [0.64, 1.21]
**Subtotal (95% CI)**		**1382**		**1342**	**100.0%**	**0.78 [0.59, 1.04]**
Total events	89		149			
Heterogeneity: $Chi^2 = 3.24$, df = 4 (P = 0.52); $I^2 = 0\%$						
Test for overall effect: Z = 1.67 (P = 0.09)						

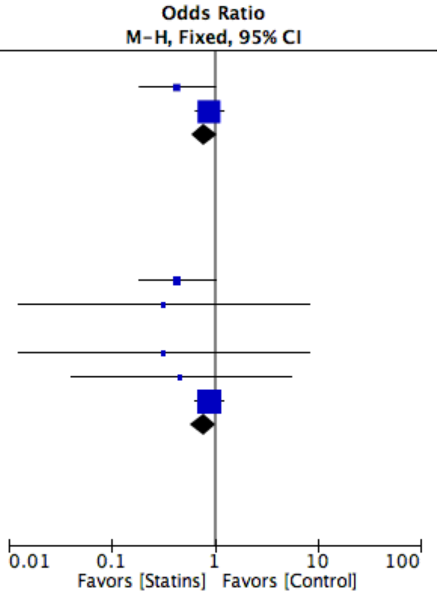

Test for subgroup differences: $Chi^2 = 0.01$, df = 1 (P = 0.91), $I^2 = 0\%$

The trial sequential analysis (TSA) for primary analysis was inconclusive. The relative risk reduction of 40% was assumed based on the clinically meaningful differences between the two treatments. The required diversity-adjusted information size is 8909 patients.

eFigure 16 – Trial Sequential Analysis: infections in low-risk of bias trials (α = 5%, β = 80%, D2 = 80.65%, RRR = 40%)

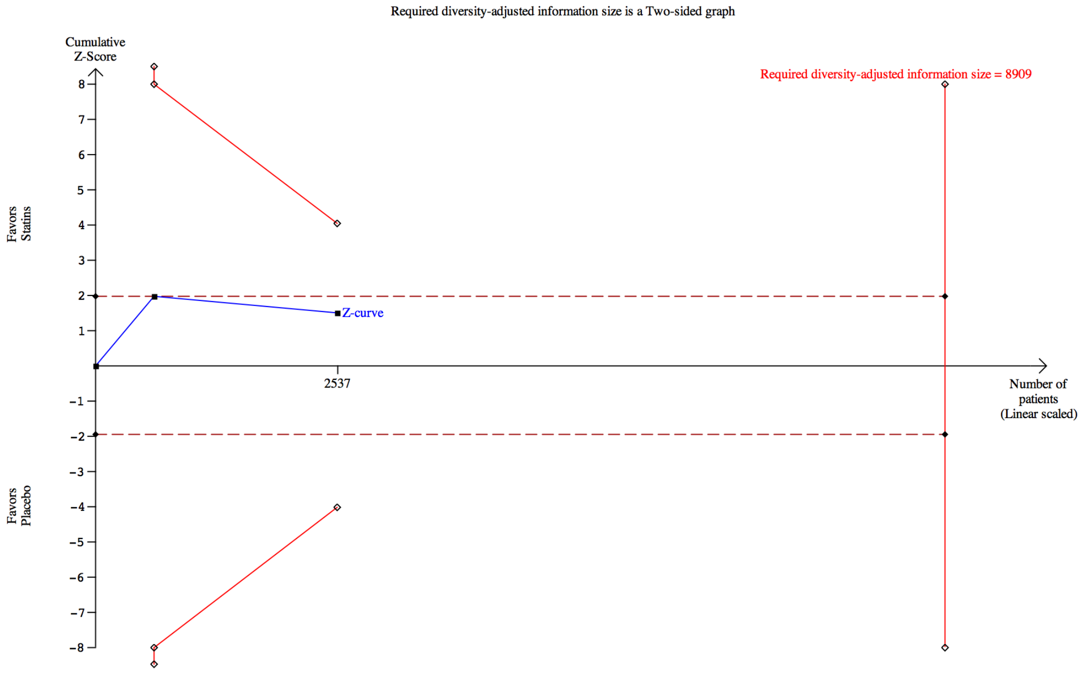

The TSA showed firm conclusion for futility when including trials with higher risk of bias

eFigure 17 – Trial Sequential Analysis: infections in low- to high- risk of bias trials (α = 5%, β = 80%, D2 = 0%, RRR = 40%)

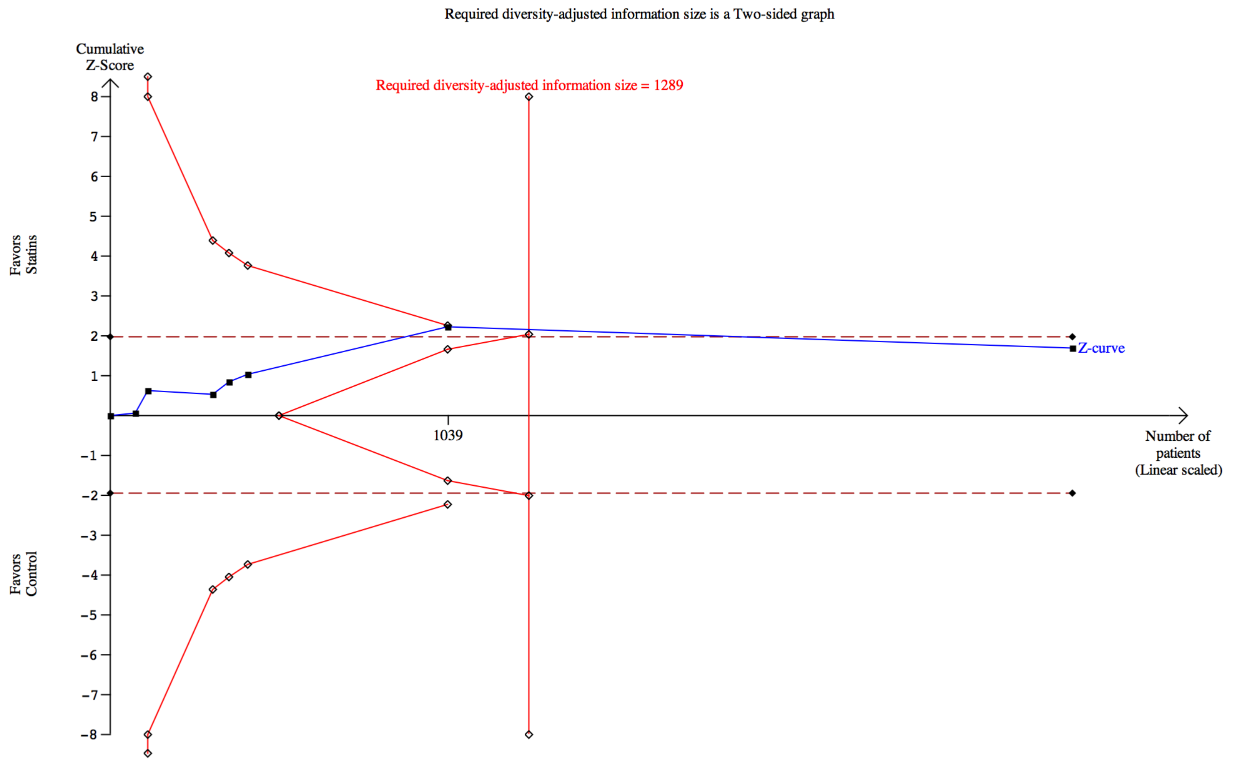

Supplemental Results: Mortality

Although 18 out of 23 trials included postoperative mortality as an outcome of interest, fatal events occurred in only 7 trials.

The administration of perioperative statins was associated with a non-significant difference in mortality among low-risk of bias trials (9 of 1318 [0.68%] in statin versus 2 of 1319 [0.15%] in the control group; OR 1.26 [95% CI, 1.05-1.52]; *p* 0.06) (Fig.6); the results did not change including higher risk of bias trials (Fig.6); TSA did not support firm evidence (*below*). The overall quality of evidence was low. The sensitivity analysis showed a significant small decrease in survival in the statin group (RD 0.01 [95% CI, 0.00-0.01]; *p* 0.04).

The trial sequential analysis (TSA) for primary analysis was inconclusive. The relative risk reduction of 80% was assumed based on the clinically meaningful differences between the two treatments in consideration of the very low incidence of the event (<1%).

eFigure 18 – Trial Sequential Analysis: stroke in low-risk of bias trials (α = 5%, β = 80%, D^2 = 0%, RRR = 80%)

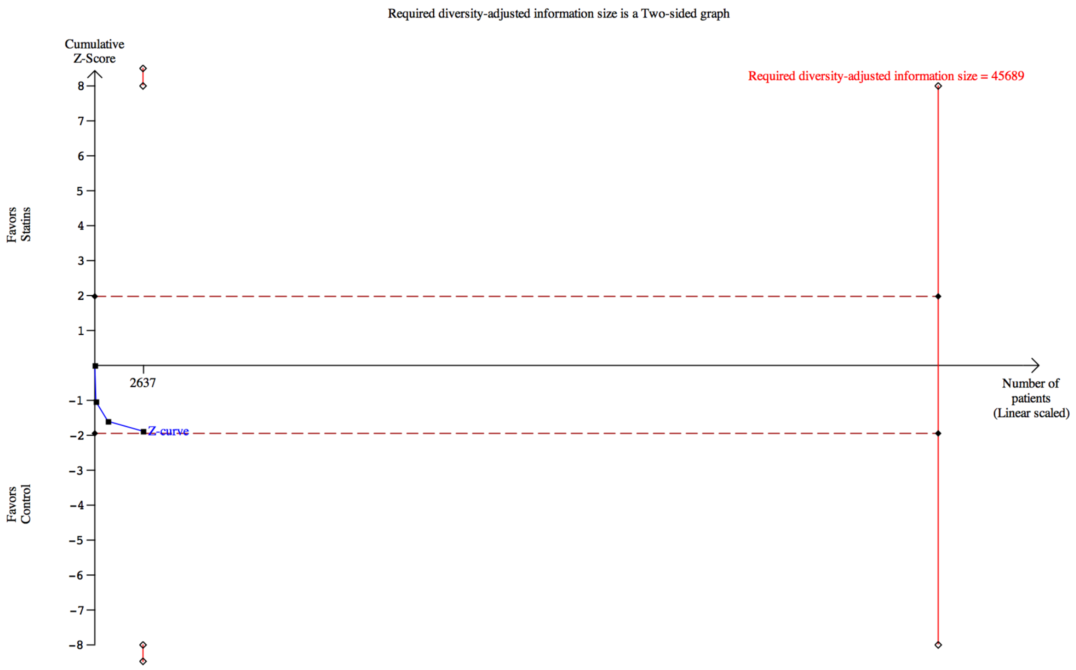

The TSA was inconclusive also when including trials with higher risk of bias.

eFigure 19 – Trial Sequential Analysis: mortality in low- to high- risk of bias trials (α = 5%, β = 80%, D^2 = 0%, RRR = 80%)

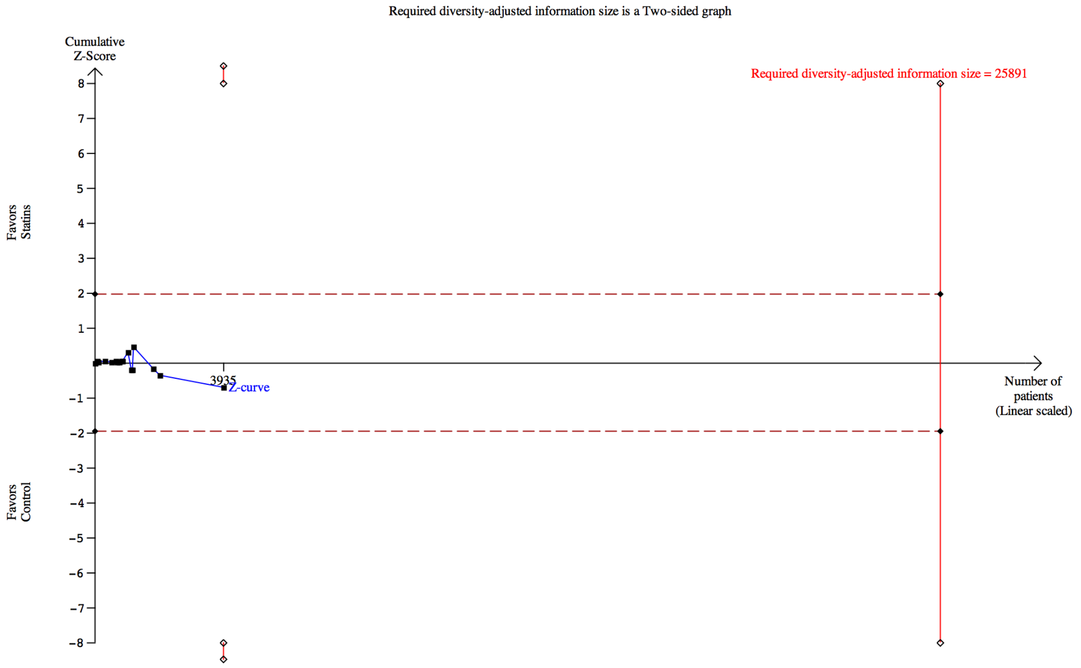

Supplemental Results: Clinical outcomes and perioperative statin regimen

Post hoc fixed-effect meta-regression analysis of low risk of bias trials failed to find possible relationships between length of preoperative regimen and clinical outcomes.

Meta-regression including low risk of bias trials failed to find any correlation. Meta-regression including all trials revealed a possible relationship between length of the preoperative regimen and AF, with patients on longer preoperative regimens presenting a lower incidence of AF than patients on shorter regimens (negative slope coefficient, p 0.024). However, we should underline that all the trials randomizing patients to longer perioperative statin therapy are at higher risk of bias.

Subgroup analysis including all trials addressing the effect of the presence or lack of postoperative regimen found significant subgroup difference for AKI (Chi^2 4.68, p 0.03) and AF (Chi^2 19.21, $p <$ 0.0001), suggesting better outcome in patients not taking postoperative statins. However, the analysis presents important limitations, since only few trials did not administer postoperative therapy and all these trials present high or unclear risk of bias.

The meta-regression was performed using Open Meta-Analyst [30,31].

Supplemental Results: Clinical outcomes in patients on chronic statin therapy or statin-naïve

Fourteen trials included only statin-naïve patients, 1 trial (30 patients) included exclusively patients on chronic statin therapy [32], 4 trials included a mixed population (1562 of 2779 [56.21%] statin-naïve patients) [28,29,33,34], and 4 trials (884 patients) did not specified population's characteristics [35–38] (Table 1).

Among low risk of bias trials, the only available outcome to estimate was AF (2 trials, 2537 patients) with no significant difference between patients statin-naïve or on chronic therapy (chi^2 0.05, p 0.82).

When including all the trials (19 trials, 4218 patients), no significant differences were found between trials including statin-naïve or mixed populations (AKI: chi^2 1.79, p 0.18; MI: chi^2 0.00, p 0.95; stroke: chi^2 0.33, p 0.56; infection; chi^2 0.76, p 0.38; mortality: chi^2 0.34, p 0.56), except for AF (chi^2 5.69, p 0.02). However, this analysis should be taken with caution, since most of the trials enrolling only statin-naïve patients are with high or unclear risk of bias.

Supplemental Results: Clinical outcomes and CABG surgery

Post-hoc fixed-effect meta-regression was employed to examine the possible influence of proportion of CABG patients on clinical outcomes. The analysis revealed that none of the outcomes were affected by length of preoperative regimen and proportion of CABG patients (positive slope coefficient, p 0.092). The meta-regression was performed using Open Meta-Analyst [30,31]

Supplemental Results: Influence of publication year and trial size on clinical outcomes

Post-hoc fixed-effect meta-regression was employed to examine the possible influence of publication year and trial size on clinical outcomes. The analysis revealed that postoperative AKI and AF were affected by both covariates (eTable 1 and 2, below). The meta-regression was performed using Open Meta-Analyst [30,31]

eTable 1 – Meta-regression of the influence of trial size on acute kidney injury and atrial fibrillation

**Postoperative outcome**	**Number of trials**	**Number of patients**	**Slope coefficient (SE)**	**P value**
Acute kidney injury				
- Low-risk of bias trials	3	2637	0.0001 (< 0.001)	0.225
- All trials	8	3354	0.0001 (< 0.001)	*0.015*
Atrial fibrillation				
- Low-risk of bias trials	2	2537	NA	NA
- All trials	18	4737	0.0001 (< 0.001)	*0.024*

SE, standard error; NA, not applicable

eTable 2 – Meta-regression of the influence of publication year on acute kidney injury and atrial fibrillation

**Postoperative outcome**	**Number of trials**	**Number of patients**	**Slope coefficient (SE)**	**P value**
Acute kidney injury				
- Low-risk of bias trials	3	2637	0.138 (0.113)	0.133
- All trials	8	3354	0.085 (0.036)	*0.017*
Atrial fibrillation				
- Low-risk of bias trials	2	2537	NA	NA
- All trials	18	4737	0.122 (0.021)	*< 0.001*

SE, standard error; NA, not applicable

Supplemental Results: Higher-risk of bias trials and clinical outcomes

eFigure 20 - Forest plot for each postoperative outcome including only trials with unclear and high-risk of bias.

Study or Subgroup	Statins Events	Statins Total	Control Events	Control Total	Weight	Odds Ratio M-H, Fixed, 95% CI
**1.7.1 Acute kidney injury: unclear and high risk of bais trials**						
Baran 2012	0	30	0	30		Not estimable
Carrascal 2016	3	47	0	43	1.4%	6.84 [0.34, 136.41]
Chello 2006	1	20	1	20	2.8%	1.00 [0.06, 17.18]
Christenson 1999	3	40	8	37	22.9%	0.29 [0.07, 1.21]
Mannacio 2008	1	100	3	100	8.8%	0.33 [0.03, 3.19]
Park 2016	21	100	26	100	61.1%	0.76 [0.39, 1.46]
Spadaccio 2010	1	25	1	25	2.9%	1.00 [0.06, 16.93]
**Subtotal (95% CI)**		**362**		**355**	**100.0%**	**0.71 [0.42, 1.20]**
Total events	30		39			
Heterogeneity: Chi^2 = 4.30, df = 5 (P = 0.51); I^2 = 0%						
Test for overall effect: Z = 1.27 (P = 0.21)						
**1.7.2 Atrial fibrillation: unclear and high risk of bais trials**						
Almansob 2012	0	68	1	64	0.6%	0.31 [0.01, 7.72]
Baran 2012	1	30	7	30	2.5%	0.11 [0.01, 0.99]
Caorsi 2008	5	21	8	22	2.2%	0.55 [0.14, 2.06]
Carrascal 2016	20	47	13	43	2.8%	1.71 [0.72, 4.08]
Castaño 2015	7	20	3	10	0.9%	1.26 [0.24, 6.45]
Chello 2006	2	20	5	20	1.6%	0.33 [0.06, 1.97]
Dehghani 2015	6	29	13	29	3.8%	0.32 [0.10, 1.02]
Ji 2009	10	71	23	69	7.3%	0.33 [0.14, 0.76]
Mannacio 2008	18	100	35	100	10.4%	0.41 [0.21, 0.78]
Melina 2009	94	315	106	317	27.0%	0.85 [0.61, 1.18]
Park 2016	42	100	50	100	10.6%	0.72 [0.41, 1.27]
Patti 2006	35	101	56	99	13.5%	0.41 [0.23, 0.72]
Song 2008	8	62	17	62	5.4%	0.39 [0.15, 0.99]
Spadaccio 2010	2	25	4	25	1.3%	0.46 [0.08, 2.75]
Sun 2011	9	49	21	51	6.1%	0.32 [0.13, 0.80]
Tamayo 2009	0	22	1	22	0.5%	0.32 [0.01, 8.25]
Vukovic 2011	4	29	11	28	3.5%	0.25 [0.07, 0.91]
**Subtotal (95% CI)**		**1109**		**1091**	**100.0%**	**0.58 [0.48, 0.70]**
Total events	263		374			
Heterogeneity: Chi^2 = 24.50, df = 16 (P = 0.08); I^2 = 35%						
Test for overall effect: Z = 5.58 (P < 0.00001)						
**1.7.3 Myocardial infarction: unclear and high risk of bais trials**						
Baran 2012	0	30	1	30	9.1%	0.32 [0.01, 8.24]
Berkan 2009	0	23	0	23		Not estimable
Carrascal 2016	2	47	0	43	3.0%	4.78 [0.22, 102.43]
Castaño 2015	0	20	1	10	11.8%	0.15 [0.01, 4.15]
Chello 2006	0	20	0	20		Not estimable
Christenson 1999	0	40	0	37		Not estimable
Dehghani 2015	0	29	0	29		Not estimable
Ji 2009	0	71	1	69	9.3%	0.32 [0.01, 7.97]
Mannacio 2008	1	100	2	100	12.2%	0.49 [0.04, 5.55]
Patti 2006	0	100	0	100		Not estimable
Song 2008	3	101	3	99	18.1%	0.98 [0.19, 4.97]
Spadaccio 2010	2	62	1	62	6.0%	2.03 [0.18, 23.02]
Sun 2011	0	25	0	25		Not estimable
Vukovic 2011	0	49	1	51	9.0%	0.34 [0.01, 8.55]
Youn 2011	0	71	3	71	21.4%	0.14 [0.01, 2.70]
**Subtotal (95% CI)**		**788**		**769**	**100.0%**	**0.64 [0.30, 1.39]**
Total events	8		13			
Heterogeneity: Chi^2 = 5.08, df = 8 (P = 0.75); I^2 = 0%						
Test for overall effect: Z = 1.12 (P = 0.26)						
**1.7.4 Stroke: unclear and high risk of bais trials**						
Baran 2012	0	30	0	30		Not estimable
Carrascal 2016	1	47	1	43	12.3%	0.91 [0.06, 15.06]
Chello 2006	0	20	0	20		Not estimable
Dehghani 2015	0	29	0	29		Not estimable
Ji 2009	0	71	1	69	18.2%	0.32 [0.01, 7.97]
Mannacio 2008	0	100	0	100		Not estimable
Park 2016	4	100	4	100	46.2%	1.00 [0.24, 4.11]
Song 2008	2	62	2	62	23.3%	1.00 [0.14, 7.33]
Spadaccio 2010	0	25	0	25		Not estimable
**Subtotal (95% CI)**		**484**		**478**	**100.0%**	**0.87 [0.32, 2.35]**
Total events	7		8			
Heterogeneity: Chi^2 = 0.43, df = 3 (P = 0.93); I^2 = 0%						
Test for overall effect: Z = 0.28 (P = 0.78)						
**1.7.5 Infection: unclear and high risk of bias**						
Chello 2006	0	20	1	20	29.9%	0.32 [0.01, 8.26]
Christenson 1999	0	40	37	0		Not estimable
Spadaccio 2010	0	25	1	25	30.0%	0.32 [0.01, 8.25]
Vukovic 2011	1	29	2	28	40.1%	0.46 [0.04, 5.43]
**Subtotal (95% CI)**		**114**		**73**	**100.0%**	**0.38 [0.07, 2.01]**
Total events	1		41			
Heterogeneity: Chi^2 = 0.05, df = 2 (P = 0.98); I^2 = 0%						
Test for overall effect: Z = 1.14 (P = 0.25)						
**1.7.7 Mortality: unclear and high risk of bais trials**						
Baran 2012	0	30	0	30		Not estimable
Castaño 2015	0	0	0	0		Not estimable
Chello 2006	0	20	0	20		Not estimable
Christenson 1999	0	40	0	37		Not estimable
Dehghani 2015	0	29	0	29		Not estimable
Ji 2009	1	100	0	100	16.7%	3.03 [0.12, 75.28]
Mannacio 2008	0	0	0	0		Not estimable
Park 2016	2	101	2	99	67.2%	0.98 [0.14, 7.10]
Patti 2006	2	50	0	50	16.1%	5.21 [0.24, 111.24]
Spadaccio 2010	0	29	0	28		Not estimable
Tamayo 2009	0	22	0	22		Not estimable
Vukovic 2011	0	29	0	28		Not estimable
Youn 2011	0	0	0	0		Not estimable
**Subtotal (95% CI)**		**450**		**443**	**100.0%**	**2.00 [0.50, 8.09]**
Total events	5		2			
Heterogeneity: Chi^2 = 0.94, df = 2 (P = 0.63); I^2 = 0%						
Test for overall effect: Z = 0.98 (P = 0.33)						

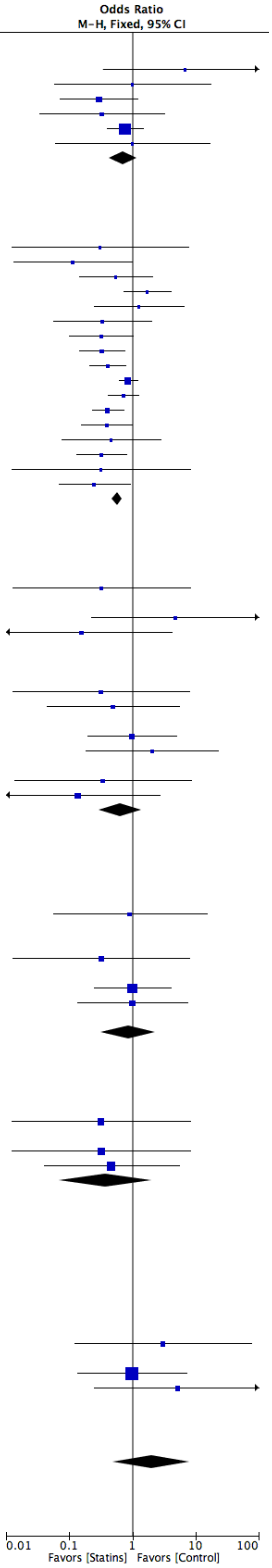

Supplemental references

1. Higgins JP, Green S. Cochrane Handbook for Systematic Reviews of Interventions. Version 5. The Cochrane Collaboration, 2011; 2011.
2. Higgins JPT, Altman DG, Gøtzsche PC, Jüni P, Moher D, Oxman AD, et al. The Cochrane Collaboration's tool for assessing risk of bias in randomised trials. BMJ. 2011;343:d5928.
3. Wetterslev J, Thorlund K, Brok J, Gluud C. Trial sequential analysis may establish when firm evidence is reached in cumulative meta-analysis. J. Clin. Epidemiol. 2008;61:64–75.
4. Brok J, Thorlund K, Gluud C, Wetterslev J. Trial sequential analysis reveals insufficient information size and potentially false positive results in many meta-analyses. J. Clin. Epidemiol. 2008;61:763–9.
5. Thorlund K, Devereaux PJ, Wetterslev J, Guyatt G, Ioannidis JPA, Thabane L, et al. Can trial sequential monitoring boundaries reduce spurious inferences from meta-analyses? Int. J. Epidemiol. 2009;38:276–86.
6. Wetterslev J, Thorlund K, Brok J, Gluud C. Estimating required information size by quantifying diversity in random-effects model meta-analyses. BMC Med. Res. Methodol. 2009;9:86.
7. Chello M, Goffredo C, Patti G, Candura D, Melfi R, Mastrobuoni S, et al. Effects of atorvastatin on arterial endothelial function in coronary bypass surgery. Eur. J. Cardiothorac. Surg. 2005;28:805–10.
8. Tamayo E, Alonso O, Alvarez FJ, Castrodeza J, Flórez S, di Stefano S. [Effects of simvastatin on acute-phase protein levels after cardiac surgery]. Med. clínica. 2008;130:773–5.
9. Lertsburapa K, White CM, Kluger J, Faheem O, Hammond J, Coleman CI. Preoperative statins for the prevention of atrial fibrillation after cardiothoracic surgery. J. Thorac. Cardiovasc. Surg. 2008;135:405–11.
10. Das S, Nanda SK, Bisoi AK, Wadhawan AN. Effect of preoperative statin therapy on early postoperative memory impairment after off-pump coronary artery bypass surgery. Ann. Card. Anaesth. 19:38–44.
11. Kourliouros A, Valencia O, Hosseini MT, Mayr M, Sarsam M, Camm J, et al. Preoperative high-dose atorvastatin for prevention of atrial fibrillation after cardiac surgery: a randomized controlled trial. J. Thorac. Cardiovasc. Surg. 2011;141:244–8.
12. Ludman AJ, Hausenloy DJ, Babu G, Hasleton J, Venugopal V, Boston-Griffiths E, et al. Failure to recapture cardioprotection with high-dose atorvastatin in coronary artery bypass surgery: a randomised controlled trial. Basic Res. Cardiol. 2011;106:1387–95.
13. Pierri MD, Crescenzi G, Zingaro C, D'Alfonso A, Capestro F, Scocco V, et al. Prevention of atrial fibrillation and inflammatory response after on-pump coronary artery bypass using different statin dosages: a randomized, controlled trial. Gen. Thorac. Cardiovasc. Surg. 2016;
14. Cannon CP, Braunwald E, McCabe CH, Rader DJ, Rouleau JL, Belder R, et al. Intensive versus moderate lipid lowering with statins after acute coronary syndromes. N. Engl. J. Med. 2004;350:1495–504.
15. Xia J, Qu Y, Yin C, Xu D. Preoperative rosuvastatin protects patients with coronary artery disease undergoing noncardiac surgery. Cardiology. 2015;131:30–7.
16. Antoniades C, Demosthenous M, Reilly S, Margaritis M, Zhang M-H, Antonopoulos A, et al. Myocardial redox state predicts in-hospital clinical outcome after cardiac surgery effects of short-term pre-operative statin treatment. J. Am. Coll. Cardiol. 2012;59:60–70.
17. Chello M, Anselmi A, Spadaccio C, Patti G, Goffredo C, Di Sciascio G, et al. Simvastatin increases neutrophil apoptosis and reduces inflammatory reaction after coronary surgery. Ann. Thorac. Surg. 2007;83:1374–80.
18. Coccia R, Spadaccio C, Foppoli C, Perluigi M, Covino E, Lusini M, et al. The effect of simvastatin on erythrocyte membrane fluidity during oxidative stress induced by cardiopulmonary bypass: a randomized controlled study. Clin. Ther. 2007;29:1706–17.
19. Dotani MI, Morise AP, Haque R, Jain AC, Gupta N, Gibson CM. Association between short-

term simvastatin therapy before coronary artery bypass grafting and postoperative myocardial blood flow as assessed by positron emission tomography. Am. J. Cardiol. 2003;91:1107–9.
20. Florens E, Salvi S, Peynet J, Elbim C, Mallat Z, Bel A, et al. Can statins reduce the inflammatory response to cardiopulmonary bypass? A clinical study. J. Card. Surg. 16:232–9.
21. Ozguler IM, Burma O, Uysal A, Akbulut H. Rosuvastatin Lowers Systemic Inflammatory Response in Coronary Artery Bypass Graft Accompanied by Cardiopulmonary Bypass Surgery: A Randomized Controlled Study. Clin. Investig. Med. Médecine Clin. Exp. 2015;38:E154-63.
22. Nakamura K, Masuda H, Kariyazono H, Arima J, Iguro Y, Yamada K, et al. Effects of atorvastatin and aspirin combined therapy on inflammatory responses in patients undergoing coronary artery bypass grafting. Cytokine. 2006;36:201–10.
23. Sasmazel A, Baysal A, Fedekar A, Buyukbayrak F, Bugra O, Erdem H, et al. The effect of statin therapy on stimulation of endothelium-derived nitric oxide before and after coronary artery bypass surgery. Heart Surg. Forum. 2010;13:E243-6.
24. Antoniades C, Bakogiannis C, Leeson P, Guzik TJ, Zhang M-H, Tousoulis D, et al. Rapid, direct effects of statin treatment on arterial redox state and nitric oxide bioavailability in human atherosclerosis via tetrahydrobiopterin-mediated endothelial nitric oxide synthase coupling. Circulation. 2011;124:335–45.
25. Antoniades C, Bakogiannis C, Tousoulis D, Reilly S, Zhang M-H, Paschalis A, et al. Preoperative atorvastatin treatment in CABG patients rapidly improves vein graft redox state by inhibition of Rac1 and NADPH-oxidase activity. Circulation. 2010;122:S66-73.
26. Mehta RL, Kellum JA, Shah S V, Molitoris BA, Ronco C, Warnock DG, et al. Acute Kidney Injury Network: report of an initiative to improve outcomes in acute kidney injury. Crit. Care. 2007;11:R31.
27. Bellomo R, Ronco C, Kellum JA, Mehta RL, Palevsky P, Acute Dialysis Quality Initiative workgroup. Acute renal failure - definition, outcome measures, animal models, fluid therapy and information technology needs: the Second International Consensus Conference of the Acute Dialysis Quality Initiative (ADQI) Group. Crit. Care. 2004;8:R204-12.
28. Billings FT, Hendricks PA, Schildcrout JS, Shi Y, Petracek MR, Byrne JG, et al. High-Dose Perioperative Atorvastatin and Acute Kidney Injury Following Cardiac Surgery: A Randomized Clinical Trial. JAMA. 2016;315:877–88.
29. Zheng Z, Jayaram R, Jiang L, Emberson J, Zhao Y, Li Q, et al. Perioperative Rosuvastatin in Cardiac Surgery. N. Engl. J. Med. 2016;374:1744–53.
30. Wallace BC, Dahabreh IJ, Trikalinos TA, Lau J, Trow P, Schmid CH. Closing the Gap between Methodologists and End-Users: *R* as a Computational Back-End. J. Stat. Softw. 2012;49:1–15.
31. Viechtbauer W. Conducting Meta-Analyses in *R* with the metafor Package. J. Stat. Softw. 2010;36:1–48.
32. Castaño M, González-Santos JM, López J, García B, Centeno JE, Aparicio B, et al. Effect of preoperative oral pravastatin reload in systemic inflammatory response and myocardial damage after coronary artery bypass grafting. A pilot double-blind placebo-controlled study. J. Cardiovasc. Surg. (Torino). 2015;56:617–29.
33. Prowle JR, Calzavacca P, Licari E, Ligabo E V, Echeverri JE, Haase M, et al. Pilot double-blind, randomized controlled trial of short-term atorvastatin for prevention of acute kidney injury after cardiac surgery. Nephrology (Carlton). 2012;17:215–24.
34. Youn Y-N, Park S-Y, Hwang Y, Joo H-C, Yoo K-J. Impact of High-Dose Statin Pretreatment in Patients with Stable Angina during Off-Pump Coronary Artery Bypass. Korean J. Thorac. Cardiovasc. Surg. 2011;44:208–14.
35. Melina G, Angeloni E, Di Nucci G, Benedetto U, Fiorani B, Sclafani G, et al. Preoperative HMG-CoA reductase inhibitors and atrial fibrillation in patients undergoing CABG: a prospective randomised trial. Eur Hear. J. 2009;30:32.
36. Almansob MAS, Xu B, Zhou L, Hu X-X, Chen W, Chang F-J, et al. Simvastatin reduces myocardial injury undergoing noncoronary artery cardiac surgery: a randomized controlled trial.

Arterioscler. Thromb. Vasc. Biol. 2012;32:2304–13.
37. Christenson JT. Preoperative lipid-control with simvastatin reduces the risk of postoperative thrombocytosis and thrombotic complications following CABG. Eur. J. Cardiothorac. Surg. 1999;15:394-9-400.
38. Caorsi C, Pineda F, Munoz C. Pravastatin immunomodulates IL-6 and C-reactive protein, but not IL-1 and TNF-alpha, in cardio-pulmonary bypass. Eur. Cytokine Netw. 2008;19:99–103.
